# Perinatal and Childhood Risk Factors of Adverse Early Childhood Developmental Outcomes: A Systematic Review Using a Socioecological Model

**DOI:** 10.3390/children12081096

**Published:** 2025-08-20

**Authors:** Kendalem Asmare Atalell, Gavin Pereira, Bereket Duko, Sylvester Dodzi Nyadanu, Gizachew A. Tessema

**Affiliations:** 1 Curtin School of Population Health, Curtin University, Perth, WA 6102, Australiagizachew.tessema@curtin.edu.au (G.A.T.); 2 College of Medicine and Health Sciences, University of Gondar, Gondar 196, Ethiopia; 3 enAble Institute, Curtin University, Kent Street, Bentley, Perth, WA 6102, Australia; 4 Research Centre for Public Health, Equity and Human Flourishing (PHEHF), Torrens University Australia, Adelaide, SA 5000, Australia; 5 Healthy Environments and Lives (HEAL) National Research Network, Canberra, ACT 2617, Australia; 6 School of Public Health, University of Adelaide, Adelaide, SA 5005, Australia

**Keywords:** developmental outcomes, early childhood, perinatal risk factors, systematic review

## Abstract

**Background:** Adverse early childhood developmental outcomes across physical, cognitive, language, communication, and socioemotional domains are major global health concerns. This systematic review aimed to synthesise perinatal and childhood risk factors using a socioecological model. **Methods:** We searched six databases for cohort, case–control, and cross-sectional studies published between January 2000 and January 2024. Studies reporting risk factors for adverse developmental outcomes were included. Findings were organised across individual, interpersonal, community, and societal levels using a socioecological model. The protocol was registered in PROSPERO (CRD42023447352). **Results:** A total of 175 studies were included. Individual-level risk factors, including preterm birth, low birth weight, male sex, chronic illness, undernutrition, and excessive screen use, were associated with adverse developmental outcomes, while exclusive breastfeeding, reading books, and storytelling were protective factors. Interpersonal risks included maternal age, education, mental health, and pregnancy complications. Community and societal risks include environmental pollution, access to education, conflict, and healthcare access. **Conclusions:** Improving early childhood developmental outcomes may require intervention at multiple levels. Future studies may need to focus on the influence of culturally and linguistically diverse backgrounds and environmental exposures on early childhood developmental outcomes.

## 1. Introduction

Globally, an estimated 250 million children fail to achieve their full developmental potential by the age of five [1]. Early childhood, defined by the World Health Organisation (WHO) as the period from prenatal development to eight years of age, is a critical window for human development [2,3]. This period lays the foundation for lifelong learning, school readiness, economic participation, and health outcomes [4,5,6]. Developmental outcomes during early childhood span across multiple domains, including physical, cognitive, language, communication, and socioemotional development [7,8,9,10]. Adverse developmental outcomes during this period, manifested as delays or difficulties in achieving developmental milestones, can lead to long-term consequences such as mental health problems, poor literacy, reduced employment opportunities, and an increased risk of involvement in criminal and violent activities [10].

A wide range of interconnected factors influence early childhood developmental outcomes, from genetic and biological characteristics to maternal health during pregnancy, child nutrition, exposure to toxic substances, accessibility and quality of healthcare services [11,12,13]. Maternal morbidity, such as hypertension, diabetes mellitus, and infectious diseases during pregnancy, can affect foetal development, which again leads to adverse childhood developmental outcomes [14,15]. Similarly, inadequate nutrition and limited access to healthcare services are associated with poor developmental trajectories [16].

Beyond individual-level determinants, broader socio-environmental contexts, including environmental pollution, neighbourhood safety, and the quality of early childhood education, critically influence developmental outcomes. These risk factors are often amplified by social inequalities and systemic barriers that disproportionately affect vulnerable populations [11,12,17,18,19,20].

Children’s optimal development is shaped by the complex interaction of biological, environmental, sociocultural, economic, political, and legal factors [21]. Given the complex and multilevel nature of these factors affecting early childhood developmental outcomes, a comprehensive approach is essential for effectively synthesising and summarising evidence from diverse and methodologically heterogeneous studies.

The socioecological model offers a flexible and structured framework for analysing risk factors at multiple levels, including individual (child), interpersonal (maternal, paternal, and household), community (school, peers, neighbourhood, and environmental factors), and societal (policy, programs, and systemic influences). Closely related to Bronfenbrenner’s ecological model, the socioecological model is widely used in public health to conceptualise interactions between social determinants of health and developmental outcomes [22]. It also guides the identification of targeted interventions to reduce developmental risks and improve outcomes.

While previous systematic reviews have examined risk factors for early childhood developmental outcomes, many have been limited by a narrow focus on single exposures or developmental domains, failing to capture the interconnected nature of these factors [18,23,24,25,26].

Grounded in the socioecological model, this systematic review examines how multilevel risk factors spanning the perinatal period to early childhood (≤8 years) influence developmental outcomes. By synthesising evidence on these exposures, the review aims to inform holistic, evidence-based strategies to prevent adverse developmental outcomes [1].

## 2. Materials and Methods

This systematic review was conducted following the Preferred Reporting Items for Systematic Reviews and Meta-Analyses (PRISMA) 2020 guidelines [27] and the Joanna Briggs Institute (JBI) manual for evidence synthesis [28,29].

### 2.1. Inclusion and Exclusion Criteria

We included observational studies (cohort, case–control, and cross-sectional) reporting the risk factors of adverse early childhood developmental outcomes. Eligibility was determined using the population, exposure, comparison, outcome, and study design (PECOS) criteria: (1) Population: The population included in this systematic review were children who underwent early childhood developmental assessments before the age of 8 years [20]. (2) Exposure: This includes perinatal and early childhood risk factors of adverse early childhood developmental outcomes, which include biological, psychosocial, behavioural, and environmental risk factors. (3) Comparator: These are children with no or low levels of exposure (if an environmental exposure) to the above risk factors. (4) Outcome: The primary outcome was adverse early childhood developmental outcomes, which include adverse outcomes of physical, cognitive, language, communication, and socioemotional development [30,31]. (5) Study design: These include observational studies such as cohort, case–control, and cross-sectional studies reporting the risk factors of adverse early childhood developmental outcomes. While there were no restrictions by geography, studies published in the English language between 1 January 2000 and 1 January 2024 were included. The year 2000 was considered due to technological change, social media use, social and cultural contexts, early childhood education, and environmental exposures over time. It is important to incorporate more recent and up-to-date knowledge in the field. There have been substantial changes in policies, interventions, and programs over the last two decades, all aimed at addressing early childhood developmental outcomes.

We excluded studies conducted exclusively among children with special health conditions such as prematurity, low birth weight, congenital anomalies, and other specific health conditions. We excluded studies such as commentaries, letters to editors, conference proceedings, case reports, case series, correspondence, descriptive statistics, interventional studies, non-human studies (e.g., animal model and in vitro), systematic review meta-analyses, and abstracts without full texts.

### 2.2. Search Strategy and Data Extraction

A comprehensive search strategy was developed collaboratively by the authors and implemented across EMBASE, PubMed, Global Health, PsycINFO, CINAHL, Web of Science Core Collection, and Google Scholar for grey literature. Search terms encompassed children’s developmental outcomes and relevant risk factors [32]. The full search terms are provided in the Supplementary Materials (Supplementary Material Table S1). The retrieved records were exported to EndNote version 20 [33], deduplicated, and exported to Rayyan [34], a systematic review management software. Initial screening (title and abstract) and subsequent full-text evaluations were primarily conducted by the first Author (KAA), with 20% independently assessed by co-authors (GAT and SDN). Data extraction, conducted by KAA using Microsoft Excel 2019 with regular discussions among the research team, included study details (author, year, country, design), sample size, age at developmental assessment, measurement tools, developmental domains, and associated risk factors. Authors were contacted for clarification or missing data when necessary.

### 2.3. Quality or Risk of Bias Assessment

Methodological quality was assessed using the updated JBI critical appraisal checklist [35,36]. Studies were classified as low, moderate, or high risk of bias based on checklist scores. Each study was rated by assigning one for yes if the paper met the criteria and zero for no/unclear if the paper did not meet the criteria listed in the JBI checklist as applied elsewhere [37].

### 2.4. Data Synthesis

Given substantial methodological outcome and risk factors measurement heterogeneity across studies, we performed a narrative synthesis informed by a socioecological model as a conceptual framework. This conceptual framework considers the complex interplay between personal, interpersonal, community, and societal factors, facilitating a multilevel understanding of risk and helping to identify the various determinants of early childhood developmental adversities. By acknowledging the interconnected factors at the individual, interpersonal, community context, and societal factors that contribute to early childhood developmental outcomes, the framework enables a comprehensive analysis essential for developing targeted interventions that address multiple layers of influences [33]. Risk factors were synthesised into individual (child-related) factors, interpersonal (family-related) factors, community factors, and societal-level influences [38,39,40]. We presented study characteristics and developmental outcomes in a tabular format, summarising the results across physical, cognitive, socioemotional, and language and communication domains. Many studies reported multiple risk factors or examined a single risk factor across multiple outcomes. The directions of association between the risk factors and outcome were assessed using the *p*-values or the confidence interval of the effect measures, such as odds ratio, relative risk, coefficient, etc.

## 3. Results

### 3.1. Study Selection and Characteristics

**Figure 1:**
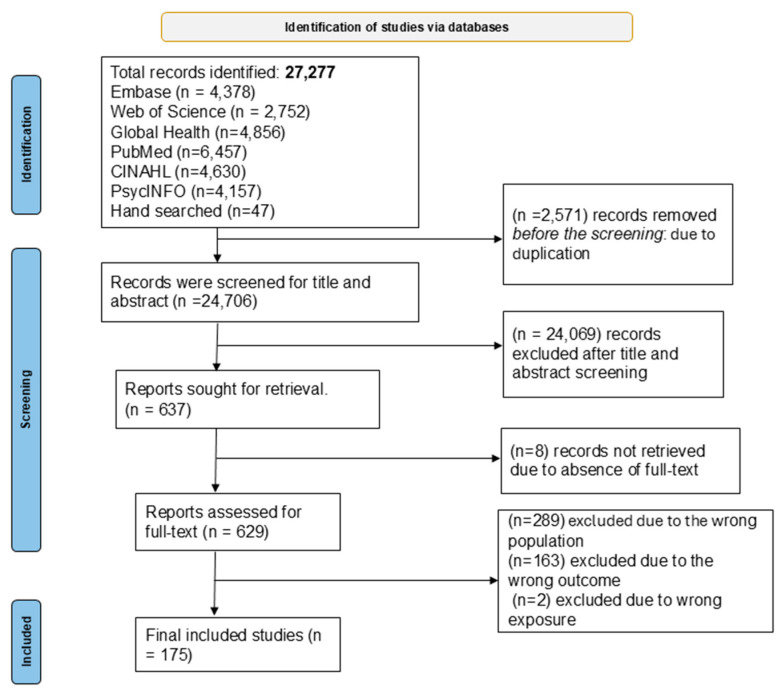
Flow diagram of investigating perinatal and early childhood risk factors for adverse early childhood developmental outcomes.

A total of 27,277 records were identified through databases and grey literature searches. After removing 2571 duplicates, 24,706 titles and abstracts were screened. Of these, 637 full-text articles were assessed for eligibility, and 175 studies met the inclusion criteria for the final review (Figure 1).

The 175 included studies were conducted in more than 80 countries. Nearly half of them (*n* = 89) originated from four countries: Australia (*n* = 33), the United States (*n* = 19), Canada (*n* = 18), and China (*n* = 17). Cohort design accounted for over three-quarters of the studies (*n* = 141). Forty-seven studies assessed childhood developmental vulnerability in multiple domains. A variety of early childhood developmental assessment tools were used. The most common was the Early Developmental Vulnerability Instrument (EDI), employed in 47 studies, followed by the Bayley Scales of Infant Development, used in 38 studies (Table 1 and Supplementary Material Table S2).

### 3.2. Adverse Early Childhood Developmental Outcomes

Nine studies [16,41,42,43,44,45,46,47,48] specifically quantified adverse developmental outcomes. In studies reporting early childhood developmental vulnerability across one or more domains, the prevalence was 28.1% in Vancouver, Canada [48], 30.2% in British Columbia, Canada [44], and 49% in Western Australia among Aboriginal children [46]. Studies from India, Kenya, and Turkey documented the prevalence of developmental delay as 6.6% [49], 16.5% [16], and 27% [42], respectively. Using the UNICEF Early Development Index, developmental vulnerability ranged from 25.1% to 34.5% in Bangladesh [41,43] and 35.0% in Nepal [45].

Across the 175 included studies, 115 examined risk factors for physical developmental vulnerability, 120 for cognitive outcomes, 90 for language and communication, and 96 for social–emotional domains; 37 studies did not specify sub-domains (Table 2).

### 3.3. Risk Factors of Adverse Early Childhood Developmental Outcomes

#### 3.3.1. Individual-Level Risk Factors

Ninety-four studies examined demographic, perinatal, health-related, nutritional, and lifestyle factors [9,11,16,31,40,41,44,45,46,48,49,50,51,52,53,54,55,56,57,58,59,60,61,62,63,64,65,66,67,68,69,70,71,72,73,74,75,76,77,78,79,80,81,82,83,84,85,86,87,88,89,90,91,92,93,94,95,96,97,98]. Males and those speaking English as a second language were consistently linked to poorer developmental outcomes, whereas two studies reported a lower risk with increasing child age. Low birth weight (8 studies), preterm birth (15 studies), and post-term birth (3 studies) showed a positive association with adverse developmental outcomes. For example, an Irish cohort study reported higher odds of childhood developmental vulnerability among low birth weight (OR = 2.6; 95% CI: 1.3, 5.0) and males (OR = 2.7, 95% CI: 1.8, 3.9) compared to their counterparts [51].

Childhood medical conditions such as infectious diseases, chronic illness, anaemia, hearing loss, plagiocephaly, untreated dental issues, childhood cancer, hospitalisation, surgery, and exposure to anaesthesia are linked with adverse developmental outcomes. Perinatal HIV exposure was associated with a marked reduction in cognitive performance, while having smaller effects on other developmental domains [81,82].

**Figure 2:**
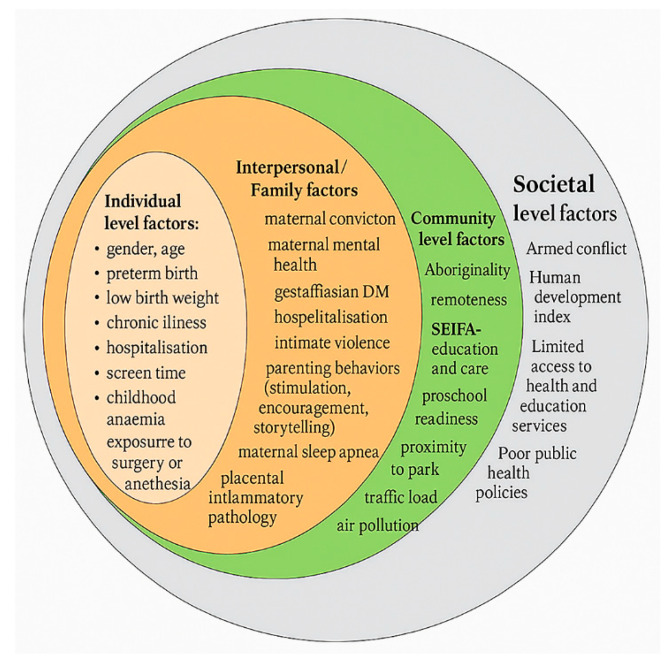
Summary of risk factors using the socioecological model at the individual, interpersonal, community, and societal levels.

Undernutrition (underweight and stunting) increased risk, whereas exclusive breastfeeding for six months and early consumption of animal-sourced foods were protective [91]. Lifestyle factors such as access to books, storytelling, iron supplementation, and deworming were beneficial, while corporal punishment, excessive screen use, physical inactivity, and inadequate sleep heighten the risk of adverse early childhood developmental outcomes (Figure 2 and Supplementary Material Table S3).

#### 3.3.2. Interpersonal and Household-Level Risk Factors

One hundred and thirty studies evaluated the maternal, paternal, and home environment influences of early childhood developmental adversities [14,15,16,41,42,43,46,47,48,49,50,51,52,53,55,63,69,90,99,100,101,102,103,104,105,106,107,108,109,110,111,112,113,114,115,116,117,118,119,120,121,122,123,124,125,126,127,128,129,130,131,132,133,134,135,136,137,138,139,140,141,142,143,144,145,146,147,148,149,150,151,152,153,154,155,156,157,158,159,160,161,162,163,164,165,166,167,168,169,170,171,172,173,174,175,176,177,178,179,180,181,182,183]. Five studies showed both young (<20 years) and advanced (>35 Years) maternal age increased risk, and eleven studies reported the protective effect of maternal education [42,46,184]. In a Canadian study, children of less educated mothers had 11-fold higher odds of developmental delays (OR: 11.12; 95% CI: 4.2, 29.3) [42]. Low socioeconomic status, consanguineous marriage, and larger sibship were also detrimental [42,48].

Among the 26 studies that assessed the link between maternal mental health and developmental adversities, 19 studies reported a positive association with poorer developmental outcomes. A study from Norway showed an elevated socioemotional risk with prenatal depression (OR: 3.4; 95% CI: 1.4, 8.0), and even higher odds with post-partum depression (OR: 3.8; 95% CI: 1.7, 8.6) [63]. Five studies [128,129,130,131] linked prolonged use of psychiatric medication during pregnancy to adverse developmental outcomes. For instance, children prenatally exposed to long-term use of Selective Serotonin Reuptake Inhibitors (SSRIS) had a significantly increased risk of fine motor developmental delays (OR: 1.5; 95% CI: 1.12, 1.94) [129].

Pregnancy complications such as anaemia, gestational diabetes, pre-eclampsia, and unhealthy behaviours such as smoking and alcohol use, extreme interpregnancy intervals, and abnormal prepregnancy BMI were linked to developmental adversities [151,153,185]. Maternal nutrition and physical activities during pregnancy play a significant role in reducing early childhood development [161].

Fifteen studies assessed prenatal exposure to insecticides, corticosteroids, mercury, and second-hand tobacco smoke with adverse developmental outcomes. Paternal conviction, mental illness, tobacco smoking, and alcohol were linked with developmental adversities, whereas higher paternal education was linked to favourable developmental outcomes in children. Positive parenting behaviours, including parental encouragement, engagement, and stimulation, reduced developmental adversities. For example, a study from China showed that having fine-motor toys at home reduced the risk of motor development issues by 67% (OR: 0.33; 95% CI: 0.22, 0.49) [180]. In contrast, poor child stimulation and low parental satisfaction were associated with an increased risk of adverse developmental outcomes. Three studies linked indoor air pollution from cooking fuels with an increased risk of childhood developmental adversities. A study by Grippo et al. found significantly higher odds of developmental adversities associated with indoor air pollution (OR: 1.3; 95% CI: 1.1, 1.53) [182].

#### 3.3.3. Community/Organisational-Level Factors

Thirty-six studies evaluated broader community influences [11,12,41,46,51,61,186,187,188,189,190,191,192,193,194,195,196,197,198,199,200,201,202]. Aboriginal children in Australia had greater developmental vulnerability, especially in social competence [40,46,61,187]. Living in remote areas and being in the lower quintiles of the socioeconomic index for areas increased developmental vulnerability. Among studies that evaluated community influences, 26 studies examined the link between air pollution exposure and adverse early childhood developmental outcomes, of which 26 studies reported increased risk. For example, children prenatally exposed to PM2.5 had higher odds of adverse developmental outcomes (OR: 1.5; 95% CI: 1.2, 2.1) [197]. Conversely, factors such as attending early childhood education and care, preschools, and being in proximity to school grounds and parks were inversely associated with developmental adversities.

#### 3.3.4. Societal, Policy/Program-Level Factors

Seven studies examined societal-level determinants [11,17,203,204,205,206,207]. Armed conflict elevated risk in low- and middle-income settings [203]. Human Development Index (HDI), and the availability of education and health services reduced early childhood developmental vulnerability. A study by Taylor et al. reported that the odds of childhood developmental vulnerability were significantly higher among children from families with low uptake of health and education services than among children from families with regular access (OR: 1.5; 95% CI: 1.2, 1.8) [205]. Canadian programs such as the Family First Home Visiting Program and the Healthy Baby Prenatal Benefit Program showed no association with developmental adversities in early childhood.

**Figure 3:**
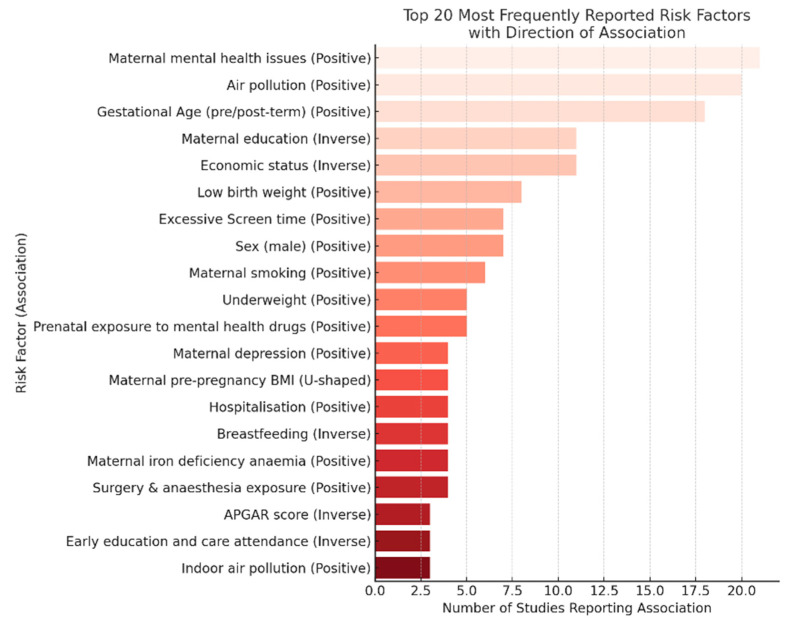
Top 20 most frequently reported risk factors affecting developmental adversities in our systematic review.

Figure 3 summarises the 20 risk factors most frequently examined across the included studies and indicates whether each factor was associated with increased (“Positive”) or decreased (“Inverse”) developmental adversities. Maternal mental health problems emerged as the most consistently reported risk factor, with 22 studies finding a positive association with developmental vulnerability. Other highly cited exposures included ambient air pollution (21 studies) and non-optimal gestational age (pre- or post-term birth; 18 studies). Several socioeconomic determinants, particularly low maternal education and low household economic status, also featured prominently, but with inverse directionality, indicating that higher education and higher income were protective.

### 3.4. Quality or Risk of Bias Assessment

**Figure 4:**
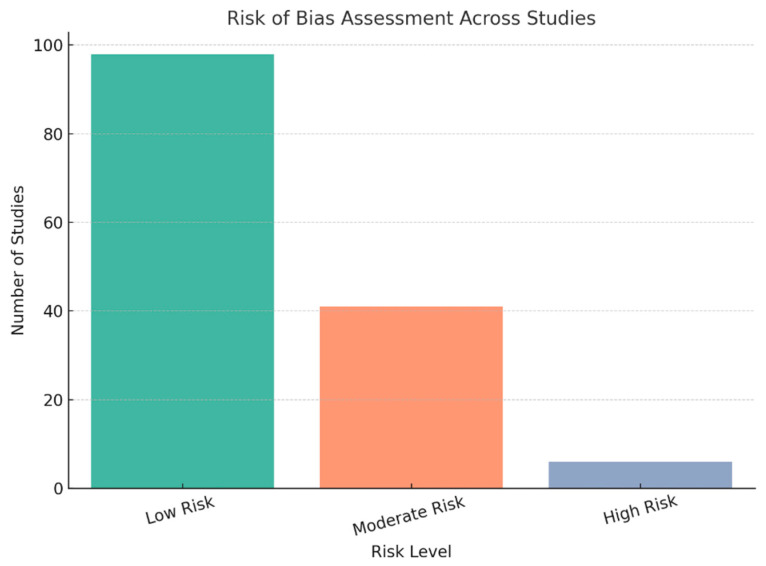
Risk of bias assessment result of 175 included studies.

Application of the JBI critical-appraisal checklists indicated that most studies were of high methodological quality. Specifically, 98 studies (70.9%) were rated as low risk of bias, 41 (26.3%) as moderate risk, and 6 (2.8%) as high risk (Figure 4 and Supplementary Material Table S4).

## 4. Discussion

This review synthesised perinatal and childhood risk factors for adverse early childhood developmental outcomes through the socioecological framework. Findings highlight the multifactorial nature of developmental adversities, spanning individual, interpersonal, community, and societal levels.

Birth characteristics such as low birth weight, prematurity, post-term birth, and male sex were consistently associated with developmental vulnerability, reflecting their impact on neural maturation and organ development [208,209,210,211,212,213]. Additional health-related risks included chronic illness, anaemia, hearing loss, plagiocephaly, untreated dental diseases, childhood cancer, and exposure to anaesthesia, which interrupts learning opportunities and or compromises neurocognitive function [16,46,53,69,70,71].

Modifiable lifestyle factors such as undernutrition, excessive screen time, physical inactivity, inadequate sleep, and punitive parenting similarly predict delays across physical, cognitive, and socioemotional development [50,214,215,216,217,218,219,220,221]. Moreover, protective factors such as exclusive breastfeeding, iron supplementation, deworming, book reading, and storytelling fostered healthy growth and language and cognitive development [91,94,181,222,223,224].

More than 80% of the included studies examined interpersonal-level factors, including maternal, paternal, and household-level influences. Extreme maternal age (<20 years or >35 years) increased risk, as did maternal mental illness, substance use, gestational complications such as anaemia, diabetes, pre-eclampsia, and short or long interpregnancy intervals [225,226,227,228,229,230,231]. Socioeconomic disadvantage, low maternal education, poverty, and single parenthood limited access to healthcare, nutrition, safe housing, and stimulating environments, whereas higher maternal education and economic stability were protective [232,233]. Paternal smoking, alcohol use, conviction, and mental illness were detrimental [234], while paternal education and active involvement in childcare supported language and socioemotional growth [235,236]. Positive parenting practices (engagement, stimulation, availability of toys) reduced risk by up to 67% [179]. Indoor air pollution from cooking fuels also emerged as a significant risk factor. In contrast, poor child stimulation and low parental satisfaction were associated with an increased risk of adverse developmental outcomes.

Remoteness, Aboriginality, and air pollution were linked to poorer developmental outcomes at the community level, reflecting structural inequities [237,238]. Conversely, participation in early childhood education and care consistently enhanced development, emphasising the value of early learning environments.

Societal-level determinants such as armed conflicts and national-level socioeconomic development (Human Development Index) also shaped early childhood outcomes [239]. Reliable access to health and education services during the first five years of life was strongly protective, whereas Canadian programs such as Family First Home Visiting and the Baby Parental Benefit programs showed no measurable effect [204,240].

Importantly, risk factors interact and compound across socioecological levels [241,242]. For example, maternal mental depression within low-income households can restrict access to healthcare and early intervention services, increasing the likelihood of adverse birth outcomes and subsequently developmental delays. Similarly, maternal smoking during pregnancy, when combined with environmental pollutants, heightens foetal exposure to neurotoxic substances and further compromises brain development.

Despite the wide range of determinants identified in this review, several critical evidence gaps remain. Research has yet to clarify how cultural and linguistic diversity (CALD) modifies developmental risk or resilience, while the cumulative effects of climate change, air pollution, and related stressors on child development are still poorly understood. Few studies map developmental vulnerability across neighbourhoods or regions, limiting the design of geographically targeted interventions. Finally, predictive modelling with routinely collected data remains underutilised, even though such approaches could facilitate earlier identification and tailored support for at-risk children.

### 4.1. Research and Policy Implications

This review identified several priority areas for future research aimed at addressing the existing evidence gaps. First, more nuanced studies are needed to explore how cultural and linguistic diversity (CALD) influences early developmental trajectories, including the identification of potential protective factors within CALD communities. Second, longitudinal studies might examine the cumulative impact of climate change and chronic exposure to environmental pollutants on neurodevelopment, acknowledging that such exposures can begin in utero and continue throughout early childhood. Third, incorporating geospatial analyses will help to identify neighbourhood-level “hot spots” of developmental vulnerability, enabling geographically targeted interventions tailored to local environmental and socioeconomic contexts. Finally, the development and validation of predictive models using routinely collected administrative and health data could facilitate early identification of children at risk of adverse developmental outcomes, enabling timely and targeted support before delays become entrenched.

Policy responses must be as multi-layered as the risk factors themselves. At the individual and family level, governments should expand universal access to high-quality antenatal care, integrate routine developmental screening into paediatric health services, and provide targeted home-visiting or parenting-support programmes for families facing psychosocial or economic disadvantage. At the community level, investment in affordable, high-quality early childhood education and care, especially in remote, low-income, and minority communities, can buffer many of the identified risks. Concurrently, local authorities should prioritise safe housing, green spaces, and clean air initiatives to reduce children’s exposure to environmental hazards. At the societal level, cross-sector policies that improve maternal education, strengthen income support, and extend paid parental leave promise long-term developmental benefits. Embedding equity metrics into health, education, and environmental policy will help direct resources to CALD and Indigenous populations, narrowing disparities in developmental outcomes.

### 4.2. Strengths and Limitations

A key strength of this review is its comprehensive and theory-informed approach, applying the socioecological model to examine a broad range of perinatal and childhood risk factors across multiple developmental domains: physical, cognitive, language and communication, and socioemotional development. This framework enables the identification of leverage points at the individual, interpersonal, community, and societal levels, offering actionable insights for policymakers and researchers addressing early childhood developmental adversity.

However, the review is constrained by notable heterogeneity in outcome measures and timing of developmental assessments. The 175 included studies utilised over 43 distinct developmental assessment tools, each with different scoring systems and age ranges, which limits direct comparability and precludes meaningful meta-analysis. Moreover, restricting the search to English-language publications may have introduced language bias, potentially underrepresenting evidence from non-English-speaking countries. Variability in data quality and reporting standards across studies further complicates cross-study comparisons. Finally, due to the heterogeneity in study designs and outcome definitions, a formal assessment of publication bias was not feasible. As a result, the findings should be interpreted with caution. Future research would benefit from greater standardisation in developmental assessment tools and reporting practices to enhance comparability and reduce bias.

## 5. Conclusions

This review shows that adverse early childhood developmental outcomes arise from a complex web of perinatal and postnatal exposures spanning individual, interpersonal, community, and societal spheres. Addressing these adversities, therefore, demands holistic, multilevel action. At the individual level, interventions should target modifiable child exposures, (e.g., nutrition, infections, and lifestyle). At the interpersonal level, programs might support maternal, paternal, and household determinants such as mental health, education, and parenting practices. Community-level strategies may expand access to quality early-childhood education, promote healthy behaviours, and improve neighbourhood environments. Finally, societal policies need to tackle structural drivers, including geographic inequities, human-development disparities, political and economic stability, and universal access to health and education services. Only an integrated approach that combines targeted support for vulnerable groups, sustained investment in early education, and robust environmental health initiatives can yield meaningful, equitable gains in children’s developmental trajectories.

## Figures and Tables

**Table 1:** Study characteristics (*n* = 175).

Study Characteristics	Number of Studies	Percent
Year of publication		
2021–2024	72	41.14
2011–2020	87	49.7
2002–2010	16	9.2
Study country		
Australia	33	18.9
Canada	19	10.9
US	20	11.4
China	17	9.7
Multi-country ***	12	6.9
Brazil	11	6.3
Spain	8	4.6
UK	7	4
Bangladesh	5	2.9
Norway	4	2.3
Others *	39	22.3
Study design		
Cohort	144	82.3
Cross-sectional	18	10.3
Survey	12	6.9
Case–control	1	0.57
Measurement tools		
Early developmental vulnerability instrument	47	26.9
Early development index	11	6.3
Bayley Scales of Infant Development	38	21.7
Age and stage questionnaire	20	11.4
Wechsler intelligence, preschool and primary scale	7	4
Others **	52	29.7

* Includes countries with studies less than 4; ** includes measurement tools used in less than 7 studies; *** Lower- and middle-income countries, Canada and Australia, Vietnam and Bangladesh, South Africa and Tanzania, and East Asia and the Pacific: Cambodia, China, Mongolia, and Vanuatu.

**Table 2:** Adverse Early Childhood Developmental Outcome (*n* = 175).

Developmental Domains and Specific Outcomes Reported in Each Study	Number of Studies
Physical development (*n* = 115 studies)	
Physical health and well-being	28
Physical development	3
Motor development	22
Gross motor development	29
Fine motor development	24
Psychomotor developmental index	9
Cognitive development (*n* = 120 studies)	
Language and cognitive	28
Cognitive development	34
Mental developmental index	13
Learning and learning disability, literacy, and numeracy	9
Problem-solving	13
Full-scale IQ	10
Verbal IQ	7
Performance IQ	6
Language and communication development (*n* = 90 studies)	
Communication and general knowledge	28
Language	27
Expressive language	10
Receptive language	9
Communication	16
Social–emotional development (*n* = 96 studies)	
Social competence	30
Emotional maturity	30
Social–emotional development	22
Personal social development	16
Unspecified subdomains (*n* = 37 studies)	
Developmental vulnerability	17
Developmental delay	20

The sum of the studies is greater than 175 since some investigated two or more developmental domains.

## Data Availability

All relevant data are included in the manuscript.

## Supplementary Materials

The following supporting information can be downloaded at: https://www.mdpi.com/article/10.3390/children12081096/s1.
