# Supplementary material for: Perinatal and Childhood Risk Factors of Adverse Early Childhood Developmental Outcomes: A Systematic Review Using a Socioecological Model
Source: Children (Basel). 2025 Aug 20;12(8):1096. doi: 10.3390/children12081096 (PMC12384675; doi:10.3390/children12081096)
Supplement: Supplementary file 1 [file children-12-01096-s001.zip › children-3749339-supplementary.pdf]

Supplementary Materials

Table S1. Search strategy

Title: Perinatal and early life risk factors of adverse child developmental outcomes: Systematic review

Databases – PubMed, Embase, Global Health, PsycINFO, CINAHL, Web of Science

	**Population:** Children	**Outcomes:** child developmental outcomes	***Exposures: P****erinatal and early life risk factors*
**Keywords**	child* OR toddler* OR preschool* OR "pre-school*" OR kindergarten* OR infant* OR baby OR babies OR “school age” OR “year five” OR “year four” OR year three” OR "early childhood" OR “school readiness”	development* OR neurodevelopment* OR “developmental outcome*” OR “developmental adversit*” OR “developmental vulnerability” OR “developmental difficult*” OR “developmental problem*” OR “development* disorder*” OR “developmental regression*” OR “developmental delay*”	*"risk factor" or "predictor" or Sociodemographic or "maternal age" or sex or income or remoteness or "Alcohol use" or "substance abuse" or Smoking or "screen time" or Drug or "early childhood education" or preterm or "gestational age" or "small for gestational age" or premature or "low birth weight" or LBW or parity or gravidity or "pregnancy complication" or preeclampsia or eclampsia or "APGAR score" or "mental health" or depression or anxiety or corticosteroid or hospitalization or malnutrition or "infectious diseases" or "chronic diseases" or hypertension or medication or anaemia or BMI or "body mass index" or Temperature or hot or heat or cold or 'climate change' or heatwave or coldwave or 'air pollution' or "particulate matter" or 'fine particle' or 'gaseous pollutant' or 'air pollutant' or "PM2.5" or PM10 or 'nitrogen dioxide' or 'sulfur dioxide' or 'sulphur dioxide' or 'nitrogen oxide' or 'carbon monoxide' or ozone or SO2 or NO2 or O3 or CO*

Table S2. Summary of included studies to investigate perinatal and early childhood risk factors of adverse early childhood developmental outcomes: systematic review and meta-analysis.

Author, Year	Objective of the study	Country	Study design	Study period	Population	Sample size	Age at developmental assessment	Developmental assessment tools	Exposure	Outcome
Duko, 2024	To estimate the causal effect of pre-eclampsia on these outcomes ascertained at the age of school entry	Australia	Cohort	2009, 2012 and 2015	Children at school entry	64391	Five years	EDI_A	Preeclampsia	✓ Social competence<br>✓ Emotional maturity
Utami, 2023	To determine the determinants of children's low cognitive development at 4–6-year-old	Indonesia	Cohort	2015-2017	Children aged 4-6 years	165	4-6 years	Wechsler Preschool and Primary Scale of Intelligence (WPPSI)	Low parental education<br>Poor child stimulation<br>Inadequate calorie intake in infancy	✓ Full-scale IQ<br>✓ Verbal IQ<br>✓ Performance IQ
Rogers, 2023	To examine the association between symptoms and diagnoses of maternal and paternal perinatal depression and anxiety with infant development	Australia	Cohort	2009-2013	Parent and children	1539	One year	Bayley Scales of Infant and Toddler Development (Bayley-III)	Maternal depression and anxiety	✓ Social-emotional<br>✓ Cognitive<br>✓ Language<br>✓ Motor<br>✓ Adaptability
Rohde, 2023	To evaluate the association between plagiocephaly and developmental delay and the timing of these diagnoses in a primary care setting, where plagiocephaly is commonly diagnosed and managed	USA	Cohort	2000-2017	Children	77,108	0-5 years	International Classification of Diseases (ICD) codes, 9th and 10th editions	Plagiocephaly	✓ Any developmental delay<br>✓ Motor delay<br>✓ Language delay<br>✓ General delay<br>✓ Social delay<br>✓ Cognitive
Smith, 2023	Does maternal mental health act as a longitudinal mediator for the association between SES and common childhood development outcomes, such as child mental health and cognitive ability	UK	Cohort	1991-1992	Mother-child dyads	13,855	1 year	Development and Well-Being Assessment (DAWBA) and Wechsler Intelligence Scale for Children, 3rd edition	Socio-economic status<br>Maternal mental Health	✓ Cognitive development
Shih, 2023	To evaluate the association between ambient particulate matter no larger than 2.5 µm in diameter (PM2.5) during the prenatal and postnatal periods and infant neurodevelopmental parameters	Taiwan	Cohort	2005	Children	17 683	0.6-1.5 years	Mandarin adaptation of the Denver Developmental Screening Test, Second Edition and the Simplified Child Developmental Screening Test in Taiwan	Air pollution: Second trimester<br>Air pollution: Third trimester	✓ Developmental delay: gross motor skills: walking with support<br>✓ Developmental delay: fine motor skills: Drawing arbitrarily.<br>✓ Developmental delay: personal social skill: Approaching when called upon.<br>✓ Developmental delay: personal social skill: Drinking with both hands.<br>✓ Developmental delay: fine motor skills: Drawing arbitrarily.

										✓ Developmental delay: personal social skill: Approaching when called upon
Bell, 2023	To compare developmental vulnerability for children whose mothers did and did not have a psychiatric hospital admission between conception and school entry in Manitoba, Canada, and Western Australia, Australia.	Canada & Australia	Cohort	2003 -2016	Children at school entry	68 274	5 years	EDI_A	Maternal mental health-related Hospital admission	✓ DV2<br>✓ DV1<br>✓ Physical health and wellbeing<br>✓ Social competence<br>✓ Emotional maturity<br>✓ Language and cognitive skills<br>✓ Communication skills and general knowledge
Wang, 2023	To evaluate the association of prenatal exposure to multiple insecticides with children's neurodevelopment and to identify critical windows of exposure	China	Prospective Cohort	2014–2017	Mother and child with two years of age	1,041	2 years	Bayley Scales of Infant Development	Organophosphate, Pyrethroid, and Neonicotinoid Insecticides	✓ Mental development delay<br>✓ Psychomotor developmental delay
Cruz-Rodríguez, 2023		Spain	Cohort	2013-2017	Mother-child dyads	434		Bayley Scales of Infant Development-III (BSID-III, cognitive, language, and motor skills)	Vitamin B12	✓ Motor developmental delay<br>✓ Gross motor<br>✓ Fine motor<br>✓ Language developmental delay
Duko, 2023	To estimate the effect (average treatment effect) of maternal prenatal tobacco smoking on childhood developmental vulnerability at age 5 years.	Australia	Cohort	2003-2015	Mother-child dyads	64,558	5 years	EDI_A	Prenatal maternal tobacco smoking	✓ DV1<br>✓ DV2<br>✓ Physical health and wellbeing<br>✓ Emotional maturity<br>✓ Social competence<br>✓ Language and cognitive skills<br>✓ Communication and general knowledge
Duko, 2023	To estimate the natural direct, indirect, and total effects of GDM on childhood developmental vulnerability in Australian Early Development Census (AEDC) domains and the proportion of the effect of GDM on early childhood developmental vulnerability in AEDC domains mediated by preterm birth in children born in Western Australia (WA).	Australia	Cohort	2003-2015	Mother-child dyads	64,356	5 years	EDI_A	Gestational diabetes mellitus	✓ DV1<br>✓ DV2
Fardell, 2023	To assess the impact of hospitalization for chronic health conditions on early child development and well-being at school start.	Australia	Cohort	2003-2012	Mother-child dyads	152,851	5 years	EDI_A	Hospitalisation with chronic condition:<br>Had hospitalisations but none with CC	✓ DV1<br>✓ Physical health and wellbeing<br>✓ Emotional maturity<br>✓ Social competence<br>✓ Language and cognitive skills<br>✓ Communication skills and general knowledge

Cruz-Rodríguez, 2023		Spain	Cohort	2013-2017	Mother-child dyads	434		Bayley Scales of Infant Development-III (BSID-III, cognitive, language, and motor skills)	Vitamin B12	✓ Motor developmental delay<br>✓ Gross motor<br>✓ Fine motor<br>✓ Language developmental delay
Grippo, 2023	To examine the associations between prenatal and postnatal indoor air pollution exposure and early childhood development in a population-based birth cohort.	USA	Cohort	2008-2010	Children	4735	1-3 years	Ages and Stages Questionnaire© (ASQ)	Indoor air pollution: cooking fuels	✓ Any fail<br>✓ Fine motor<br>✓ Gross motor<br>✓ Communication<br>✓ Personal Social<br>✓ Problem-solving
Islam, 2023	To estimate the Early Childhood Development Index (ECDI) of 3- and 4-year-old children in Bangladesh and examine the relationship between maternal parity and early childhood development	Bangladesh	Survey	2019	Children aged 3-5 years	9453	3-5 years	EDI	Maternal parity	✓ Early Development Index<br>✓ Physical<br>✓ Literacy-numeracy<br>✓ Learning<br>✓ Social-emotional
Kennedy, 2023	To determine the causal effect of IVF conception on primary school-age childhood developmental and educational outcomes, compared with outcomes following spontaneous conception.	Australia	Cohort	2005-2014	Children at school entry	173,200	Five years	EDI_A	IVF	✓ DV2<br>✓ Physical health and wellbeing<br>✓ Social competence<br>✓ Emotional maturity<br>✓ Langauge and cognitive skills<br>✓ Communication skills and general knowledge
Kim, 2023	To investigate these relationships in infants aged 36–42 months	South Korea	Cohort	2018–2022	Mother-child dyads	221	Three years	Bayley scales of infant development; the Swanson, Nolan, and Pelham rating scale (SNAP); and the Child Behavior Checklist (CBCL)	Chemicals: MEHP, mono (2-ethylhexyl) phthalate<br>Chemical: MiNP, mono-isononyl phthalate<br>Chemical: PFNA, perfluorononanoic acid<br>Chemical: PFOS, perfluorooctane sulfonic acid	✓ Motor<br>✓ Social-emotional<br>✓ Adaptive area
Le˜ao, 2023		Brazil	Cohort	2004	Mother-child dyads	3787	Four years	Battelle Development Inventory	Screen time: TV at 2 years<br>Screen time: TV at 4 years<br>Change in TV<br>Other screens at 4 years<br>Total screen at 4 years	✓ Child development
MORROW, 2003		USA	Cohort	1990-1993	Infants	476	0.5-3 years	Bayley Scales of Infant Development, scored using the Kent Scoring Adaptation for language	Prenatal Cocaine Exposure	✓ Language development
Odo, 2023		LMIC	Survey	2011-2018	Children	57,647	3-4 years	EDI	PM2.5: 5 µg/m increase	✓ Cognitive developmental delay
Pokharel, 2023	To investigate the relationship between the number, frequency, and cumulative consumption of ASF at 6, 9, 12, and 18 months of age and ECD outcomes at 24	Nepal	Cohort	2015-2019	Mother-child dyads	701	2 years	Ages-and-Stages Questionnaire (ASQ-3)	Animal-source food consumption	✓ Developmental outcome

	months of age, controlling for physical growth									
Reardon, 2023	To examine associations between prenatal exposure to 25 PFAAs, including perfluorooctane sulfonate (PFOS) and perfluorooctanoate (PFOA) isomers, and child neurodevelopment among 490 mother-child pairs in a prospective Canadian birth cohort, the Alberta Pregnancy Outcomes and Nutrition (APrON) study	Canada	Cohort	2009-2012	Children	490	2 years	Bayley Scales of Infant and Toddler Development, 3rd Edition (Bayley-III)	Mercury and perfluoroalkyl acid isomers	✓ Cognitive<br>✓ Language<br>✓ Motor<br>✓ Social-emotional<br>✓ Adaptive
Rivero, 2023		Spain	Cross-sectional study		Parent and children	90	2-3 years	Bayley Scales of Infant Development-III (BSID-III)	Parenting behaviour: mother's encouragement<br>Parenting behaviour: Father encouragement	✓ Composite language<br>✓ Receptive language<br>✓ Expressive language<br>✓ Composite language<br>✓ Receptive language<br>✓ Expressive language
Ahmed, 2022	To explore the role of multiple exposure periods to ambient particulate matter with diameter <2.5 μm (PM2.5) and nitrogen dioxide (NO2) on emotion and behaviour and early development in children <13 years.	Australia	Cohort	2016/17	Mother and their children	5471	7 years	Strengths and Difficulties Questionnaire; SDQ	Distance to major roads + exposure to window periods close to birth.<br>Child lifetime road distance	✓ Emotional and behavioural problems<br>✓ Communication delay<br>✓ Gross motor delay
Basnet, 2022	To examine the association of maternal resources for care on child length, motor development and language development of children 12–23.9 months old.	Vietnam and Bangladesh	Survey	2010	Mothers and their youngest children 12–23.9 months old	1438	1-2 years	Language development was assessed using 21 and 20 items in Bangladesh and Vietnam, Motor development was measured using 29 items in both countries	Maternal resources for care: Well-nourished	✓ Motor developmental delay<br>✓ Language developmental delay
Cao, 2022	This survey was conducted to find out the association between the home environment and the early development of infants	China	Cohort	2014-2015	Infants aged 3-11 months	1850	1 year	The Age and Stage Questionnaire-Chinese (ASQ-C)	Home environment: physical space,<br>variety of stimulations,<br>fine motor toy and<br>gross motor toy	✓ Communication developmental delay<br>✓ Gross motor<br>✓ Fine motor<br>✓ Problem-solving<br>✓ Personal Social developmental delay
Chen, 2022	To examine whether placental inflammatory pathology is associated with subsequent child neurodevelopment	USA	Cohort	1959-1976	Children aged 24 months	54371	1 year	Bayley Scales of Infant Development	Placental inflammatory pathology	✓ Motor delay<br>✓ Mental scale delay<br>✓ IQ
Hao, 2022	To investigate the joint effect of maternal pre-pregnancy body mass index (BMI) and gestational weight gain (GWG) on children's cognitive development.	China	Cohort	2013-2014	Mother-child dyads	1685	at the mean age of 55.6 months	Wechsler Preschool and Primary Scale of Intelligence-Fourth Edition	Maternal prepregnancy BMI: <18.5 with excessive gestational weight gain<br>as compared to 18.5-24.9	✓ Full-scale IQ
Hasan, 2022	To investigate the status of early childhood development (ECD) and its associated	Bangladesh	Survey	2012-2019	Children aged 3-5 years	17,494	3-5 years	EDI_UNICEF	Child aged four years as compared to three years	✓ Child on Track 2012

	factors. Additionally, aimed to compare the changes of significantly associated factors using two multiple indicator cluster surveys (MICS) in Bangladesh.							Female<br>Maternal education in secondary and higher<br>Early childhood education attendance<br>Book reading at home<br>Toys<br>Child punishment	
Hua, 2022	To examine the long-term neuropsychological outcomes of children born in a full range of GA with a nationally representative sample in Chin	China	Cohort	2018–2019	Children aged 3-5 years	149 909	3-5 years	The Ages and Stages Questionnaires-Third Edition (ASQ-3)	Gestational age at birth:<34 (very and moderately preterm) as compared with 39-40<br>Gestational age at birth:34–36 (late–preterm) as compared with 39-40<br>Gestational age at birth:37–38 (early–term) as compared with 39-40<br>Gestational age at birth:41 (late-term) as compared with 39-40<br>Gestational age at birth:>41 (post–term) as compared with 39-40	✓ Communication<br>✓ Gross motor<br>✓ Fine motor<br>✓ Problem-solving<br>✓ Personal and Social Behaviour
Kerai, 2022	To assess the association between screen time and child developmental vulnerability	Canada	Cohort	2019	Children at school entry	2818	Five years	EDI_C	Screen time> 1 hour per day<br>Annual household income:<75,000<br>Physical activity: not participated<br>Sleep: <10 hour<br>Sex: male	✓ Physical health and wellbeing<br>✓ Social competence<br>✓ Emotional maturity<br>✓ Language and cognitive skills<br>✓ Communication skills and general knowledge
Ma, 2022	To investigate parents' engagement, children's screen time, and their social competence among Taiwanese Chinese children's families at three-time points	Taiwan	Cohort	2016-2019	Taiwanese Chinese children	1,785	3-5 years	Child's Social Competence Development Questionnaire developed by the KIT project	Screen time<br>Parental engagement<br>Family socioeconomic status<br>From year three to year 5 using parental engagement	✓ Social competence
Burger, 2022	To investigate the neurodevelopment of infants at 6 months post-term with exposure to mothers with a clinical diagnosis of persistent mental health disorders residing in low-income communities in Cape Town, South Africa	South Africa	Cohort	2014-2019	Mother-child dyads	97	0.5 year	Bayley Scales of Infant and Toddler Development (BSID-III)	Maternal mental health<br>Maternal mental health: psychotic disorder	✓ Cognitive composite score<br>✓ Motor composite<br>✓ Language<br>✓ Social-emotional<br>✓ Adaptive behaviour
Moreno-Giménez, 2022	To examine the association between maternal age and offspring neurodevelopment in terms of both	Spain	Cohort	2010-2011	Mother-child dyads	101	2 years	Ages & Stages Questionnaires-Third edition	Maternal age	✓ Communication<br>✓ Fine motor<br>✓ Gross motor

	psychomotor development (Ages & Stages Questionnaires-3) and emotional competencies (Early Childhood Behavior Questionnaire)								✓ Problem Solving<br>✓ Personal Scoial
Motoki, 2022	To investigate and examine the impact of GWG on infant neurodevelopmental abnormalities at 12 months of age using the data of a nationwide Japanese cohort study	Japan	Cohort	2011-2014	Infants	30,694	1 year	Ages and Stages Questionnaire, third edition (ASQ-3)	Gestation weight gain (GWG): Below GWG reference guideline<br>Gestation weight gain (GWG): Above GWG reference guideline	✓ Developmental delay: communication<br>✓ Developmental delay: Gross motor<br>✓ Developmental delay: Fine motor<br>✓ Developmental delay: Problem-solving<br>✓ Developmental delay: Personal Social
Rana, 2022	To examine the associations between HAP and early childhood development (ECD) outcomes among children under 5 years of age in Bangladesh and explore potential effect modification by sex and urbanicity	Bangladesh	Survey	2019	Children	9395	3-5 years	EDI	Household Air pollution	✓ Total developmental delay<br>✓ Socioemotional<br>✓ Learning cognitive
Rithipukdee, 2022	To investigate the suspected delay in language development and the factors associated with the suspected delay in language development among early childhood in Southern Thailand	Thailand	Cross-sectional study	2020-2021	Children aged 2-5 years	425	2-5 years	Child's development with the Developmental Surveillance and Promotion Manual (DSPM)	More than 2 hours of screen time<br>Moderate to difficult temperament	✓ Language developmental delay
Rocha, 2022	To evaluate the association of parenting practices with child development in a cross-sectional population-based study in a low-income state in northeastern Brazil	Brazil	Cross-sectional study	2017	Mother-child dyads	3566	1-5 years	Ages and Stages Questionnaire Version 3	Positive parenting behaviours: SMD for each additional parenting behaviour	✓ Communication<br>✓ Gross motor<br>✓ Fine motor<br>✓ Problem-solving<br>✓ Personal Social
Roy, 2022	To investigate the effect of antenatal and postnatal depression on child development at 12 months of age	India	Cohort	2019	Mother-child dyads	174	1 year	Developmental Assessment Scale in Indian Infants (DASII)	maternal depression: antenatal depression	✓ Developmental delay
Russell, 2022	To explore the associations between the frequency of early stimulating activities between the caregiver and the child, dietary knowledge, and child development outcomes in Zanzibar, Tanzania	Tanzania	Cross-sectional study	2019	Mother-child dyads	500	1.5-2 years	CREDI tool	Caregiver/mother interaction or stimulation activities<br>Knowledge of dietary diversity (four or more food groups<br>compared to fewer than four)	✓ Overall score<br>✓ Cognitive<br>✓ Language<br>✓ Social-emotional<br>✓ Motor
Shrestha, 2022	To assess the association of stunting, wasting and underweight (three prominent forms of malnutrition) with the four domains of the ECD index (literacy-numeracy, physical, social-emotional and learning development) among children 36–59 months of age	Nepal	Cross-sectional study	2019	Children	2871	3-5 years	EDI	Malnutrition: Stunting<br>Malnutrition: underweight	✓ Developmentally on Track<br>✓ Literacy-numeracy<br>✓ Physical development<br>✓ Learning development<br>✓ Literacy-numeracy<br>✓ Physical development<br>✓ Learning

Shuffrey, 2022	To examine associations between prenatal maternal depression and anxiety with child social-emotional and cognitive development in a cohort from the Western Cape Province of South Africa	South Africa	Cohort	2014-2015	Maternal-infant dyads	600	3 years	Brief Infant- Toddler Social Emotional Assessment and the Bayley Scales of Infant Development III Screening Test (BSID- III ST)	Maternal depression and anxiety	✓ Social-emotional problem<br>✓ Mean cognitive score
Tao, 2022	to investigate whether maternal antenatal corticosteroid administration was associated with neurodevelopment in infants at 1 year of age	China	Cohort	2018-2020	Pregnant mother and their children until 1 year	1759	1 year	Bayley Scales of Infant and Toddler Development, Third Edition	Corticosteroid: dexamethasone exposure during pregnancy	✓ Non-competent cognitive domain
Turunç, 2022		LMIC	Survey	2010-2017	Family	16,010	3-5 years	EDI	Domestic violence justification: maternal justification<br>Domestic violence justification: paternal justification	✓ Early childhood developmental score
Urizar, 2022		USA	Cohort	1998-2001	Mother-child dyads	96	1-5 years	Mullen Scales of Early Learning: AGS Edition (MSEL) for cognitive and Vineland Social-Emotional Early Childhood Scales (VSEEC) for social-emotional	Maternal depression	✓ Cognitive development<br>✓ Social-emotional development
Wang, 2022	To evaluate and compare the associations of prenatal and early postnatal exposure to ambient particulate matter with offspring neurodevelopment at 2 years of age and to identify which period was more sensitive to the effects of ambient particulate matter on offspring neurodevelopment	China	Cohort	2013-2014	Mother-child dyads	1331	2 years	Bayley Scale of Infant Development (BSIDCR)	PM2.5 during pregnancy<br>PM2.5 during the first two years after birth<br>PM10 Pregnancy<br>PM10 during the first two years after birth	✓ MDI<br>✓ PDI
Yu, 2022	To explore the effects of prenatal maternal exposures to air SO2/ NO2 on toddler neurodevelopment and the potential effect modification by ambient temperature in Shanghai, China	China	Cohort	2010	Mother-child dyads	225	2-3 years	A Chinese version of GDS	Prenatal exposure to ambient air pollution: SO2	✓ Gross motor<br>✓ Fine motor<br>✓ Adaptive behaviour<br>✓ Social behaviour<br>✓ Language
Dhamrait, 2022		Australia	Cohort	2009–2015	Children at school entry	32,324	5 years	EDI_A	Post birth Interpregnancy Interval < 6 month as compared to 18-23 months<br>Post birth Interpregnancy Interval 6-11 months month as compared to 18-23 months	✓ DV1<br>✓ DV2
Lindquist, 2022	To investigate the association between elective birth at 39weeks' gestation and the risk of childhood developmental vulnerability	Australia	Cohort	2005-2018	Children at school entry	88165	5 years	EDI_A	Elective birth at 39 weeks gestation	✓ DV2<br>✓ Physical health and wellbeing<br>✓ Social competence<br>✓ Emotional maturity<br>✓ Language and cognitive skills

										✓ Communication skills and general knowledge
Whitten, 2022	To examine the associations between early life exposure to DVA and early childhood developmental vulnerability	Australia	Cohort	2009	Children at school entry	82,501	5 years	EDI_A	Any domestic violence	✓ Physical health and wellbeing<br>✓ Social competence<br>✓ Emotional maturity<br>✓ Language and cognitive skills<br>✓ Communication skills and general knowledge
Taylor, 2021	To investigate patterns of health and education service use from birth through kindergarten (age four years), the associations with cumulative risks, and developmental vulnerability in the first year of full-time school (age five years)	Australia	Cohort	2015	Children at school entry	5,440	5 years	EDI_C	Health and education services before 5 years: declining use as compared to regular use<br>Health and education services before 5 years: low use as compared to regular use	✓ DV
Morris, 2021	To investigate the impact of early childhood cancer on developmental outcomes	Australia	Cohort	2009, 2012, and 2015	Children at school entry	241	5 years	EDI_A	Childhood cancer	✓ DV<br>✓ Physical health and wellbeing<br>✓ Social competence<br>✓ Emotional maturity<br>✓ Language and cognitive skills<br>✓ Communication skills and general knowledge
Dhamrait, 2021	To investigate the associations between interpregnancy intervals (IPIs) and developmental vulnerability in children's first year of full-time school (age 5).	Australia	Cohort	2009, 2012 or 2015	Children at school entry	34 574	5 years	EDI_A	IPIs of <6 months relative to IPIs of 18–23 months<br>IPIs of 24-59 months relative to IPIs of 18–23 months<br>IPIs of 60-119 months relative to IPIs of 18–23 months<br>IPIs of >=120 months relative to IPIs of 18–23 months	✓ DV1<br>✓ DV2
Enns, 2021	to determine the impact of the Healthy Baby Prenatal Benefit on birth and early childhood outcomes among Manitoba First Nations women and their children	Canada	Cohort	2003–2011	First Nation children at school entry	8209	5 years	EDI_C	Healthy Baby Prenatal Benefit	✓ DV1<br>✓ Physical health and wellbeing<br>✓ Social competence<br>✓ Emotional maturity<br>✓ Language and cognitive skills<br>✓ Communication skills and general knowledge
Wei, 2021	To characterize the bidirectional associations of the term-born infants' neurodevelopment in five domains and physical growth in early life.	China	Cohort	2017-2020	Mother-child dyads	688	0.6-1 year	Development in neurological outcomes (Gesell Development Scale)	BMI z-scores	✓ Adaptive behaviour<br>✓ Gross motor<br>✓ Fine motor<br>✓ Language<br>✓ Social behaviour
Wang, 2021	To explore the association between prenatal ambient fine particulate matter (PM2.5) exposure and early childhood neurodevelopment in a large birth cohort study of4009 maternal-child pairs	China	Cohort	2016-2018	Mother-child dyads	4009	0.6-2 years	ASQ®, 3rd edition (ASQ®-3)	PM2.5 exposure during pregnancy<br>PM2.5 exposure during pregnancy: 10-μg/m3 increase in PM2.5 24 months	✓ Overall score<br>✓ Communication<br>✓ Gross motor<br>✓ Fine motor<br>✓ Problem solving<br>✓ Personal-social

Wallenborn, 2021	To investigate the importance of breastfeeding for early childhood development in the modern urban context of São Paulo, Brazil.	Brazil	Cohort	2012- 2014	Mother-child dyads	2288	3 years	Regional Project on Child Development Indicators (PRIDI) (Engle scale) and HAZ, Strengths and Difficulties Questionnaire (SDQ), Caregiver Reported Early Development Instruments (CREDI)	Exclusive breastfeeding	✓ Cognitive and social-emotional development
Varadarajan, 2021	To determine the burden of ST, associated sociodemographic factors, and its impact on domains of child development.	India	Cross-sectional study	2019	Children under 5 years	718	0.5-6 years	Communication DEALL Developmental Checklist	Screen time: excessive use	✓ Any one domain<br>✓ Two or more domain<br>✓ Three or more domain<br>✓ Receptive or expressive langue delay<br>✓ Language or social interaction delay<br>✓ Any communication delay
Ma, 2021	To investigate the associations between prenatal cadmium exposure and neurodevelopment in 2-year-old children and examine the influence of mother/child characteristics.	Japan	Cohort	2011–2014	Mother-child dyads	3545	2 years	Kyoto Scale of Psychological Development (KSPD)	Prenatal cadmium exposure: Maternal blood cadmium<br>Prenatal cadmium exposure: cord blood cadmium	✓ Postural motor<br>✓ Cognitive adaptive<br>✓ Language Social<br>✓ Developmental quotient
Dhamrait, 2021	To quantify the risk of developmental vulnerability for children at school entry, associated with gestational age at birth and to understand the impact of sociodemographic and other modifiable risk factors on these relationships	Australia	Cohort	2003-2015	Mother-child dyads	64,810	5 years	EDI_A	Gestational age: extremely preterm <28 weeks as compared to term	✓ DV1<br>✓ DV2
Gao, 2021	To assess the associations of iron supplementation and deworming separately or combined with improved early childhood development (ECD) status.	LMIC	Cross-sectional study	2011-2018	Children age 3-5 years	29,729	3-5 years	Early Childhood Development Index (ECDI)	Iron supplementation and deworming	✓ Early Childhood Development Index z score<br>✓ Early Childhood Development Index z score: literacy numeracy<br>✓ Early Childhood Development Index z score: learning<br>✓ Early Childhood Development Index z score: socioemotional<br>✓ Early Childhood Development Index z score: physical
Gleason, 2021		USA	Cohort	1959-76	Live singleton birth	39 19	1-4 years	Bayley Scales of Infant Development at 8 months, Stanford–Binet IQ (SBIQ) domains and seven years using the Wechsler Intelligence Scales for Children (WISC) and Wide-Range Achievement Tests (WRAT).	Gestational age at birth: 37 as compared to 40	✓ Bayley mental<br>✓ Bayley Psychomotor<br>✓ Full-scale IQ<br>✓ Verbal IQ<br>✓ Performance IQ<br>✓ Stanford–Binet IQ

Goto, 2021	To elucidate the relationship between exposure to conflicts and ECD.	LMIC	Cross-sectional study	2011-2018	Children age 3-5 years	27538	3-5 years	EDI	Armed conflict: one year<br>Armed conflict: 2 years<br>Armed conflict: 3 years<br>Armed conflict: 4 years<br>Armed conflict: 5 years	✓ Being developmentally on track
Allel, 2021	To analyse the association between exposure to ambient fine particulate matter (≤2.5 µm [PM2.5]) and cognitive development indicators in a cross-sectional analysis of children (aged 3–4 years) in 12 LMICs	LMIC	Cross-sectional study	2010-2018	Children aged 3-5 years	57647	3-5 years	UNICEF's Early Childhood Development Index (ECDI) questionnaire	PM2.5	✓ Cognitive developmental delay
Bornstein, 2021	To understand how country-level indicators of human development, captured as life expectancy, education, and income, are related to children's development extending beyond survival and physical health to literacy and numeracy, socioemotional development, and approaches to learning.	LMIC	Cross-sectional study	2009-2017	Children aged 3-5 years	159 959	3-5 years	Early Childhood Development Index	Human development index	✓ Developmental vulnerability
Jang, 2021	To characterise the association between maternal pregnancy intention and socioemotional developmental outcomes in a Brazilian sample of preschool-aged children	Brazil	Cohort	2016	Children age 4-5 years	1,034	4-5 years	Ages & Stages Questionnaires: Social-Emotional, Second Edition (ASQ: SE-2) screening tool	Unintended pregnancy	✓ Social-emotional<br>✓ Internalizing Behaviors<br>✓ Externalizing Behaviors<br>✓ Internalizing and Externalizing behaviors<br>✓ School Readiness
Jarvis, 2021	To investigate the association between lifetime residential exposure to greenspace and early childhood development and evaluate the extent to which reductions in traffic-related air pollution and noise mediate this association	Canada	Cohort	2000-2005	Children at school entry	37 745	5 years	EDI_C	NO2<br>PM2.5<br>Day-evening-night noise level	✓ DV
Rocha, 2021	To evaluate the association of screen exposure with child communication, gross motor, fine-motor, problem-solving, and personal-social development scores	Brazil	Cross-sectional study	2017	Mother-child dyads	3155	1-5 years	Ages and Stages Questionnaire Version 3	Screen time: all types<br>Screen time: TV<br>Screen time: Interactive media	✓ Communication<br>✓ Gross motor<br>✓ Fine motor<br>✓ Problem-solving<br>✓ Personal Social
Rocha, 2021	To assess the association between pregnancy and neonatal factors with children's developmental scores in a cross-sectional, population-based study of children in Ceará, Brazil	Brazil	Cross-sectional study	2017	Mother-child dyads	3566	1-5 years	Ages and Stages Questionnaire Version 3	Birth weight: < 2500g<br>Birth weight: Folic acid supplementation.<br>Birth weight: extended NICU or incubator use	✓ Communication<br>✓ Gross motor<br>✓ Fine motor<br>✓ Problem-solving<br>✓ Personal Social
Gupta, 2021	To find out the prevalence and correlates of developmental delay among children under two years of age in slums of Burdwan Municipality, West Bengal	India	Cross-sectional study	2019	Children	240	2 years	EDI_A	Maternal education: less than primary<br>Socio-economic status<br>Birth weight: low birth weight	✓ Developmental delay

Zheng, 2021	we aimed to describe patterns of neurodevelopmental outcomes among preschoolers who experienced the Flint water crisis before age 2 years	USA	Cohort	2012-2014	Mother-child dyads	170	2 years	Comprehensive assessment battery to evaluate skills	Maternal IQ	✓ Developmental delay
Zhong, 2021	to investigate the interrelationships between the caregiver's mental health, parenting practices, and the child's ECD outcomes in rural China	China	Cohort	2016	Mother-child dyads	1787	0.5-2 years	Bayley Scales of Infant Development version III	Caregiver DASS-21	✓ Cognitive<br>✓ Language<br>✓ Motor<br>✓ Socioemotional
Taylor, 2020	To investigate the associations between clusters of early life risk factors and developmental vulnerability in children's first year of full-time school at age	Australia	Cohort	2008– 2010/ 2015	Children at school entry	5440	5 years	EDI_A	Sociodemographic and health behaviour risk<br>Teenage mother and sociodemographic risk<br>Birth risk groups<br>Birth, sociodemographic and health behaviours	✓ DV
Simpson, 2020	To investigate developmental vulnerability for Australian children with reported HL, as measured by teachers completing the AEDC	Australia	Cohort	2015	Children at school entry	285 232	5 years	EDI_A	Hearing loss	✓ DV1
Singal, 2020	To determine if in-utero selective serotonin reuptake inhibitor (SSRI) or selective serotonin-norepinephrine inhibitor (SNRI), exposure is associated with developmental vulnerability in kindergarten among children whose mothers were diagnosed with prenatal mood or anxiety disorder	Canada	Cohort	1996 and 2014, 2015	Mother-child dyads	3050	5 years	EDI_C	Utro antidepressant exposure	✓ DV1<br>✓ DV2<br>✓ Physical health and wellbeing<br>✓ Social competence<br>✓ Emotional maturity<br>✓ Language and cognitive skills<br>✓ Communication skills and general knowledge
Drago, 2020		South Africa and Tanzania	Cohort	2009-2017	Mother and child with two years of age	401	5 years	Wechsler Preschool Primary Scales of Intelligence (WPPSI)	Psychosocial and environmental determinants: organization of the environment, opportunity for stimulation, socioeconomic status, provision of play material, avoidance punishment, maternal depressive symptoms, maternal education	✓ Full-scale IQ<br>✓ Verbal IQ<br>✓ Performance IQ
Gül, 2020	To evaluate the relationship between maternal psychiatric symptoms and developmental delay types of infants who do have not a known risk factor and are expected to show healthy development but have some behavioural and developmental problems.	Turkey	Cohort		Children aged 27- 42 months	79	2-4 years months	Ankara Developmental Screening Inventory (ADSI)	Maternal Paranoid ideation<br>Maternal anxiety<br>Maternal hostility<br>Maternal psychoticism<br>Infant competence domain<br>Maternal depression	✓ Language cognitive delay<br>✓ Gross motor delay<br>✓ Gross motor delay<br>✓ Social and self-help delay<br>✓ Fine motor delay<br>✓ Total developmental delay

Jeong, 2020	to examine the association between IPV victimisation and ECD and assess whether the association was mediated by maternal and paternal stimulation.	LMIC	Survey	2011-2018	Children age 3-5 years	15 202	3-5 years	EDI	Intimate partner violence: any<br>Intimate partner violence: physical<br>Intimate partner violence: emotional<br>Intimate partner violence: sexual	✓ Early Childhood Development Index
Strobel, 2020	To determine the associations of these high-need profiles with the likelihood of a child becoming developmentally vulnerable	Australia	Cohort	2009, 2012	Children at school entry	2715	5 years	EDI_A	Male<br>Preterm birth: <37<br>Birth weight <2500<br>Contact with child protection at least once<br>Siblings >= 3<br>Maternal age less than 20<br>Mental health<br>Very remote<br>Socioeconomic status: most disadvantage<br>Child hospitalization: >=2 days<br>Maternal hospitalization: >=4	✓ DV1
Van der Veere, 2020	To determine the effect of prenatal exposure to SSRIs on children's cognitive, motor, and behavioural outcomes at 2.5 years, adjusted for maternal depression and anxiety	Netherlands	Cohort	2007-2010	Mother child dyads	111	2.5 years	Bayley Scale of Infant and Toddler Development, 3rd Edition	Maternal SSRI exposure	✓ Cognition<br>✓ Gross motor<br>✓ Fine motor
Wall-Wieler, 2020	To assess the relation between exposure to maternal depression before age 5 and 5 domains of developmental vulnerability at school entry, overall, and by age at exposure	Canada	Cohort	2005-2016	Children	52103	5 years	EDI_C	Maternal depression	✓ DV1<br>✓ Physical health and wellbeing<br>✓ Social competence<br>✓ Emotional maturity<br>✓ Language and cognitive skills<br>✓ Communication skills and general knowledge
Webb, 2020	To examine whether the association between neighbourhood SES and developmental health outcomes of Canadian kindergarten children is different for girls than for boys	Canada	Cohort	2007-2013	Children at school entry	315,811	5 years	EDI_C	Socio-economic status<br>Male<br>Socio-economic status and Male	✓ DV1<br>✓ Physical health and wellbeing<br>✓ Social competence<br>✓ Emotional maturity<br>✓ Language and cognitive skills<br>✓ Communication skills and general knowledge
Collier, 2020	To extend this previous work quantifying the magnitude of socioeconomic inequalities in child development across jurisdictions, and explores how levels of developmental vulnerability and socioeconomic inequalities in development	Australia	Census	2009, 2012, 2015 and 2018	Children at school entry	1,162,076	5 years	EDI_A	Jurisdiction/Jurisdictional socio-economic inequality in 2009<br>Jurisdiction/Jurisdictional socio-economic inequality in 2018	✓ DV1

	have changed over time since the first national census in 2009 to its most recent collection in 2018									
Leonard, 2020	To investigate the association of anaemia between ages six and 23 months with indicators of childhood development at school age among children of remote Aboriginal and Torres Strait Islander communities of Far North Queensland	Australia	Cohort	2012, 2015	Children who have hemoglobin tests between 6 and 23 months	250	Five years	EDI_A	Child anemia when age between 6 and 23 months<br>Mother anemic before and during pregnancy<br>Mother smoking<br>Male	✓ DV2
Ha, 2019	To investigate whether proximity to major roadways, or prenatal and early-life exposures to PM2.5 and O3 increases the risk of early developmental delays	USA	Cohort	2008-2010	Children	5825	3 years	Ages and Stages Questionnaire (ASQ)	Distance to major road < 50 meter as compared to >1000	✓ Any domain<br>✓ Fine motor<br>✓ Gross motor<br>✓ Communication<br>✓ Personal-social<br>✓ Problem solving
Janus, 2019	To evaluate the relationship between UDNs and developmental health among kindergarten children using the Early Development Instrument (EDI).	Canada	Cohort	2010-2015	Children at school entry	576,264	5 years	EDI_C	Unaddressed dental need	✓ DV<br>✓ Physical health and wellbeing<br>✓ Social competence<br>✓ Emotional maturity<br>✓ Language and cognitive skills<br>✓ Communication skills and general knowledge
Smith-Nielsen, 2019	To investigated whether PPD was associated with infant social withdrawal during interaction with a tester in a psychological test situation and whether infant social withdrawal in the test situation mediated the association between PPD and infant cognitive scores reported in a previous study	Copenhagen	Cohort	2016	Children of mothers with postpartum depression and their controls	69	3-5 years	Bayley-III	Maternal depressive symptoms	✓ Cognitive development
Wang, 2019	To investigate the nutritional status and gross motor development of children in a suburban area of northeast China and the factors influencing these issues	China	Cross-sectional study	2017	Mother-child dyads	189	1-3 years	Maternal report based on WHO multicentre growth reference- study	Birth weight: >= 4000<br>Exclusive breastfeeding	✓ Delayed walking >=15
Lertxundi, 2019	To analyse the associations between prenatal PM2.5 and NO2 exposure and cognitive functions in children at 4–6 years of age, including sex differences and the modification effect of the duration predominant breastfeeding these associations	Spain	Cohort	2004-2008	Children age 4–6 years	1119	4 years	McCarthy Scales of Children's Abilities (MSCA)	Prenatal exposure to PM2.5<br>Prenatal exposure to NO2	✓ General Cognitive Index<br>✓ Verbal IQ<br>✓ Perceptive-Manipulative<br>✓ Numeric<br>✓ Memory<br>✓ Motor<br>✓ Fine<br>✓ Gross
Guo, 2019	To evaluate the associations of both prenatal and early childhood CPF exposure with neurodevelopment of children	China	Cohort	2009-2013	Children aged 3 years	498	3 years	Gesell Developmental Schedules	Chlorpyrifos exposure: I unit increase in transformed TCPy concentrations: prenatal exposure	✓ Motor area<br>✓ Adaptive area<br>✓ Language area<br>✓ Social area

										✓ Motor area<br>✓ Adaptive area<br>✓ Language area<br>✓ Social area
Williamson, 2019	To explore the association between child, family and area-level characteristics associated with developmental vulnerability, amongst Aboriginal and non-Aboriginal children in their first year of school	Australia	Cohort	2009 or 2012	Children at school entry	86388	5 years	EDI_A	Aboriginality	✓ Social competence<br>✓ Emotional maturity
Razaz, 2019	To investigate the associations between Apgar scores at 1 and 5 min, across the entire range of score values, and child developmental health at 5 years of age.	Canada	Cohort	1993-2014	Children at school entry	150 081	5 years	EDI_C	One minute APGAR score 2 as compared to 10<br>Five minutes APGAR score 1 as compared to 10	✓ DV
O'Leary, 2019	To examine the association between surgical procedures that require general anesthesia before primary school entry and child development in biological siblings	Canada	Cohort	2004-2012	Children at school entry	10 897	5 years	EDI_C	Surgery and general anesthesia exposure	✓ DV1<br>✓ Physical health and wellbeing<br>✓ Social competence<br>✓ Emotional maturity<br>✓ Language and cognitive skills<br>✓ Communication skills and general knowledge
Peterson, 2019	To examine the effects of IPV and the quality of the home environment on children's language development at age 3 years	USA	Cohort	2017	Mother child dyads	79	3 years	Peabody Picture Vocabulary Test, Fourth Edition<br>Expressive Vocabulary Test, Second Edition	IPV	✓ Receptive language<br>✓ Expressive language
Polte, 2019	To assess the association between traffic-related air pollution and cognitive function in a prospective birth cohort in Rome	Norway	Cohort	2008-2010	Mother-child dyads	1336	2 years	Ages & Stages Questionnaire: Social-Emotional (ASQ: SE)	Prenatal anxiety	✓ Social-emotional development problem <50
Rao, 2019	To examined associations between participation, intensity (hours per week), duration (months attended), and total dosage (total hours attended) in early childhood education (ECE) and children's cognitive, language, and socio-emotional development in Cambodia, China, Mongolia, and Vanuatu using data from the validation sample of the East Asia-Pacific Early Child Development Scales (EAP-ECDS)	East Asia and the Pacific: Cambodia, China, Mongolia, and Vanuatu	Survey	2013–2014	Children	4712	3-6 years	The East Asia Pacific Early Child Development Scales (EAP-ECDS)	Early childhood education	✓ Cognitive development<br>✓ Language and emergent literacy<br>✓ Socio-emotional development
Widen, 2019	To examine sex-specific associations between prepregnancy BMI, GWG, and child neurodevelopment at age 7	USA	Cohort	1998-2006	Mother and child who are African American and Dominican women	368	7 years	Wechsler Intelligence Scale for Children (WISC-IV)	Maternal prepregnancy BMI: overweight for boys<br>Maternal prepregnancy BMI: obese for boys<br>Maternal prepregnancy BMI: overweight for girls	✓ Full-scale IQ

									Maternal prepregnancy BMI: obese for girls	
Wu, 2018	To compare developmental outcomes between young children who were HIV-exposed but uninfected (HEU) and their HIV-unexposed and uninfected (HUU) peers in rural Yunnan, China	China	Cohort	2010-2013	HIV-exposed and unexposed children	500	0.6-3 years	Bayley Scales of Infant and Toddler Development–Third Edition (Bayley III)	HIV exposure	✓ Cognitive problem<br>✓ Language<br>✓ Motor<br>✓ Social-emotional<br>✓ Adaptive behaviour
Bell, 2018	To examine the early developmental outcomes of children of convicted parents.	Australia	Cohort	2003 -2009	Children at school entry	19071	5 years	EDI_A	Parental conviction<br>Community order and incarnation	✓ Physical health and wellbeing<br>✓ Social competence<br>✓ Emotional maturity<br>✓ Language and cognitive skills<br>✓ Communication skills and general knowledge
Bai, 2018	To examine the correlation between birth season and early childhood development	China	Cohort	2013	Mothers with infants 8-10 months	650	1 year	Bayley Scales of Infant Development	Born season	✓ Mental development delay<br>✓ Psychomotor developmental delay
Chaudhury, 2018	To compare neurodevelopmental outcomes between HIV-exposed/ uninfected (HEU) children exposed in utero to 3-drug combination antiretroviral therapy (ART) versus zidovudine (ZDV) monotherapy	Botswana	Cohort	2010-2012	Children aged 24 months	598	2 years	Bayley Scales of Infant and Toddler Development Third Edition (Bayley-III)	Exposed to ART Vs ZDV	✓ Cognitive developmental delay<br>✓ Gross motor delay<br>✓ Fine motor delay<br>✓ Expressive language delay<br>✓ Receptive langue delay
Demirci, 2018	To identify risk factors of developmental delay	Turkey	Nested case-control	2013-2014	Children	285	1-5 years	ASQ-TR	Maternal age >=35<br>Maternal education primary and lower<br>Maternal age secondary<br>Consanguineous marriage<br>Type of delivery	✓ Developmental delay
Falster, 2018	To quantify a child's risk of developmental vulnerability at age five, according to their mother's age at childbirth	Australia	Cohort	2003-2012	Mother-child dyads	99,530	5 years	EDI_A	Maternal age: 15–<30 years<br>Maternal age 35-45 years	✓ DV1<br>✓ Physical health and wellbeing<br>✓ Emotional maturity<br>✓ Social competence<br>✓ Language and cognitive skills<br>✓ Communication skills and general knowledge
Green, 2018	To investigate the effects of in-utero exposure to maternal infection and non-infectious diseases during pregnancy on offspring developmental vulnerabilities at age 5 years, in the context of estimated effects for early childhood exposures to infectious and non-infectious diseases and maternal mental illness.	Australia	Cohort	2003-2009	Children at school entry	66,045	5 years	EDI_A	Maternal infection during pregnancy<br>Maternal non-infectious disease during pregnancy<br>Maternal infection post-pregnancy (child age ≤4 years)<br>Maternal non-infectious disease post pregnancy (child age ≤4 years)	✓ Physical health and wellbeing<br>✓ Social competence<br>✓ Emotional maturity<br>✓ Language and cognitive skills<br>✓ Communication and general knowledge

									Child infection (child age ≤4 years)<br>Child non-infectious disease (child age ≤4 years)	
Hanly, 2018	To quantify how gestational age relates to developmental vulnerability in both populations	Australia	Cohort	2009 or 2012	Children aged 27- 42 months	97 989	5 years	EDI_A	Early or moderate preterm <=33 as compared to full term 39-40<br>Late term 34-36 as compared to full term 39-40.<br>Early term 37-38<br>Late-term 41 weeks as compared to 39-40)<br>Post-term >=42<br>Aboriginal	✓ DV
He, 2018		China	Cross-sectional study	2017	Mother and children age less than 2 years	446	2 years	Bayley Scales of Infant Development III (BSID-III)	Environmental tobacco smoke	✓ Cognitive score<br>✓ Language score<br>✓ Motor score<br>✓ Social-emotional score
McCormack, 2018	To examine associations between prenatal alcohol use by both mothers and their partners and infant cognitive developmental outcomes at 12 months	Australia	Cohort	2008-2013	Parent and children	1331	1 year	Bayley Scales of Infant Development − Third edition	Maternal alcohol use first tirmisiter: low<br>Maternal alcohol use first trimester: moderate<br>Maternal alcohol use first tirmisiter: Binge<br>Maternal alcohol use first tirmisiter: heavy<br>Maternal alcohol use second tirmisiter<br>Maternal alcohol use third tirmisiter<br>partner alcohol use third: low<br>partner alcohol use third: moderate<br>partner alcohol use third: binge<br>partner alcohol use third: heavey	✓ Cognitive outcome
Mora, 2018	To examine whether prenatal mancozeb exposure and excess Mn were associated with neurodevelopment in 355 1-year-old infants living near banana plantations with frequent aerial mancozeb spraying in Costa Rica.	Costa Rica	Cohort	2010-2011	Infant age 1 year	355	1 year	Bayley Scales of Infant and Toddler Development, 3rd edition (BSID-III)	ETU<br>MnH	✓ Cognitive<br>✓ Language<br>✓ Motor<br>✓ Social-emotional

Neamah, 2018	To examine these relationships among women and their children in Tanzania	Tanzania	Survey	2010-2014	Children aged 18-36 months	1031	1.5-3 years	Bayley Scales of Infant Development, Third Edition (BSID-III)	Maternal depression<br>IPV: sexual<br>IPV: physical<br>IPV: physical and sexual	✓ Cognitive<br>✓ Receptive language<br>✓ Expressive language<br>✓ Motor
ROUX, 2018	To assess the neurodevelopment of breastfed HIV-exposed uninfected (HEU) and breastfed HIV-unexposed (HU) children in the context of universal maternal antiretroviral therapy (ART)	South Africa	Cohort	2013–2016	HIV-exposed and unexposed children	521	1.5 years	Bayley Scales of Infant and Toddler Development, Third Edition (BSID-III)	HIV exposure	✓ Cognitive<br>✓ Motor<br>✓ Language
Gonzalez, 2017	To analyse the association between maternal depressive symptoms at 6 months after birth and a child's neurodevelopmental disorders at 18 months old in a homogeneous population characterized by a low socioeconomic and cultural level	Uruguay	Prospective Cohort	2010-2012	Mother-child dyads	127	1.5 years	Brunet Lézine test	Postnatal depression	✓ Developmental delay
Chartier,2017	To examine the effectiveness of Families First Home Visiting (FFHV) for decreasing rates of being in the care of child welfare, decreasing hospitalizations for maltreatment-related injuries, and improving child development at school entry	Canada	Cohort	2003–2009	Children at school entry	9746	5 years	EDI_C	Family First Home Visiting program	✓ Physical health and wellbeing<br>✓ Social competence<br>✓ Emotional maturity<br>✓ Language and cognitive skills<br>✓ Communication skills and general knowledge
Berglund, 2017	To assess the impact of maternal Iron deficiency and overweight on neurodevelopment	Spain	Cohort	2008-2012	Mother child dyads	331	1.5 years	Bayley Scales of Infant Development, Third Edition (BSID-III).	Maternal Iron deficiency during pregnancy<br>Maternal overweight/obesity during pregnancy	✓ Language developmental delay<br>○ Expressive<br>○ Receptive<br>✓ Motor developmental delay<br>○ Gross motor<br>○ Fine motor<br>✓ Cognitive developmental delay<br>✓ Socioemotional developmental delay
O'Neill, 2017	We examined the effect of neonatal body composition and customised birthweight centiles on neurocognitive and behavioural outcomes at age 2	Ireland	Cohort	2009-2011	Children age 24 months	254	2 years	Bayley Scales of Infant and Toddler Development Version III (BSID-III)	Tin for gestational age	✓ Developmental delay
Polanska, 2017	to examine the relationship of maternal prenatal and postnatal anxiety with the social-emotional development of 2-year-old children while taking into account a comprehensive number of potential confounders	Poland	Cohort	2007	Mother-child dyads	461	2 years	Bayley Scales of Infant and Toddler Development (Bayley 3rd edition)	Environmental Tobacco Smoke Exposure during Pregnancy	✓ Cognitive<br>✓ Language<br>✓ Motor
Bin, 2017	To assess whether in-utero exposure to maternal sleep apnea is associated with	Australia	Cohort	1994–2012 or AEDC 2009 and 12	Children at school entry	626 188	5 years	EDI_A	Maternal sleep apnea	✓ DV1

	long-term childhood consequences is unclear								
Christian, 2017	To determine if a child's social, emotional and physical development is associated with the neighbourhood-built environment.	Australia	Census	2012	Children at school entry	23395	5 years	EDI_A	Aboriginal or Torres Strait<br>Distance to the nearest park<br>Distance to the nearest school ground	✓ Physical health and wellbeing<br>✓ Social competence<br>✓ Emotional maturity
Junge, 2017	To investigate if maternal depression at different time points during the perinatal period impacts children's social-emotional development at two years of age.	Norway	Cohort		Mother-child dyads	1235	Two years	Ages and Stages Questionnaire: Social-Emotional	Perinatal depression: 32 gestation weeks<br>Perinatal depression: 8 weeks postpartum<br>Perinatal depression: both occasions<br>Current depression/2 years postpartum<br>Preterm birth	✓ Social, emotional score > 50
Warshafsky, 2016	To prospectively examine whether children of women with a pregnancy affected by severe pre-eclampsia (PE), compared to children of women without a PE-affected pregnancy, have differences in neurodevelopmental performance up to 5 years of age	Canada	Prospective Cohort	2003-2009	Mother-child dyads	269	1-5 years	Ages and Stages Questionnaire (ASQ)	Severe pre-eclampsia	✓ Developmental delay
Porta, 2016	To assess the association between traffic-related air pollution and cognitive function in a prospective birth cohort in Rome	Italy	Cohort	2003-2004	Children	474	7 years	Wechsler intelligence Scale for children-iii	Air pollution: NO2 (10 µg/m3)<br>Air pollution: PM2.5 (10 µg/<br>Air pollution: Total Traffic L	✓ Full scalee IQ<br>✓ Verbal scale IQ<br>✓ Performance scale IQ
Kariuki, 2016	To examine the effects of hospitalisation for early-life infection on early childhood development	Australia	Cohort	2009	Children at school entry	87 026	Five years	EDI_A	Hospital admission for infection: admitted to hospital with an infection<br>Hospital admission for infection: admitted to hospital with an infection once<br>Hospital admission for infection: admitted to hospital with an infection two or more	✓ DV1<br>✓ DV2<br>✓ Physical health and wellbeing<br>✓ Social competence<br>✓ Emotional maturity<br>✓ Language and cognitive skills<br>✓ Communication and general knowledge<br>✓
Graham, 2016	To impact general anaesthesia on specific neurodevelopmental domains, using the Early Development Instrument (EDI) assessment administered in kindergarten	Canada	Cohort	2006, 2007, 2009, and 2011	Children at school entry	18,056	5 years	EDI_C	General anaesthesia: single exposure<br>General anaesthesia: multiple exposure	✓ DV<br>✓ Physical health and wellbeing<br>✓ Social competence<br>✓ Emotional maturity<br>✓ Language and cognitive skills<br>✓ Communication and general knowledge
Bell, 2016	To evaluate the association of prenatal exposure to multiple insecticides with	Australia	Cohort	2003 -2009	Children at school entry	22 890	5 years	EDI_A	Chronic illness	✓ Physical health and wellbeing<br>✓ Social competence

	children's neurodevelopment and to identify critical windows of exposure									✓ Emotional maturity<br>✓ Language and cognitive skills<br>✓ Communication skills and general knowledge
Ballantyne, 2016	To compare the risk of developmental delay between late preterm and full-term Canadian-born infants at age 12 months and to determine infant and maternal factors associated with the risk of delay	Canada	Prospective Cohort	2008-2010	Mother-child dyads	208	1 year	Ages and Stages Questionnaires 3rd edition (ASQ-3)	Late preterm 34-37	✓ Gross motor developmental delay<br>✓ Communication delay
Handal, 2016	To estimate the association between prenatal exposure to selective serotonin reuptake inhibitors (SSRIs) and motor development in children considering the effect of maternal symptoms of anxiety and depression before, during and after pregnancy.	Norway	Cohort	1999-2008	Mother and children	51404	3 years	Ages and Stages Questionnaire (ASQ)	Selective serotonin reuptake inhibitors	✓ Fine motor developmental delay
Ghassabian, 2016	To examine whether children of mothers with a medical condition diagnosed before or during pregnancy took longer to achieve gross motor milestones up to age 24 months.	USA	Cohort	2008-2010	Mother-child dyads	4909	1-2 years	Maternal report after giving training	Gestational DM<br>Gestational Hypertension	✓ Gross motor: sitting without support.<br>✓ Gross motor: walking with assistance<br>✓ Gross motor: walking with assistance
Richards, 2016	To describe early childhood cognitive ability and kindergarten academic achievement across gestational age at birth in a population-based longitudinal cohort	USA	Cohort	2006-2007	Children	6150	2 years	Bayley Short Form-Research Edition (BSF-R) Mental Scale, an abbreviated version of the Bayley Scales of Infant Development Second Edition (BSID-II) developed for the ECLS-B	Gestational age: Early preterm (22-27 weeks) as compared to Term (39-40 weeks)<br>Gestational age: Moderate preterm (28-33 weeks) as compared to Term (39-40 weeks)<br>Gestational age: Late preterm (34-36 weeks) as compared to Term (39-40 weeks)<br>Gestational age: Early term (37-38 weeks) as compared to Term (39-40 weeks)<br>Gestational age: Late term (41 weeks) as compared to Term (39-40 weeks)<br>Gestational age: Post term (42 weeks) as compared to Term (39-40 weeks)	✓ Metal scale score
O'Leary, 2016	To investigate whether surgery in early childhood is associated with adverse effects	Canada	Cohort	2004-2012	Children at school entry	84276	5 years	EDI_C	Exposed to surgery	✓ DV<br>✓ Physical health and wellbeing<br>✓ Social competence

	on child development measured at primary school entry									✓ Emotional maturity<br>✓ Language and cognitive skills<br>✓ Communication skills and general knowledge
Razaz, 2016	To association between parental multiple sclerosis (MS) and parental MSrelated clinical factors on developmental health	Canada	Cohort	1994-2011	Children at school entry	3771	5 years	EDI_C	Parental Multiple Sclerosis<br>Maternal multiple sclorosis<br>Father multiple sclerosis	✓ DV1
Pearce, 2016	To investigate whether children who do not have a healthy BMI are more likely to be developmentally vulnerable on a global measure of child development at the start of school	Australia	Cohort	2009	Children at school entry	7533	5 years	EDI_A	Thin<br>Overweight<br>Obese	✓ DV1<br>✓ Physical health and wellbeing<br>✓ Social competence<br>✓ Emotional maturity<br>✓ Language and cognitive skills<br>✓ Communication skills and general knowledge
Razaz, 2016	To assess the relationship between the 5 min Apgar score and developmental vulnerability at 5 years of age.	Canada	Cohort	1999-2011	Children at school entry	33 883	5 years	EDI_C	Five minutes APGAR score 0-6 as compared to APGAR score 9-10	✓ Physical health and wellbeing<br>✓ Social competence<br>✓ Emotional maturity<br>✓ Language and cognitive skills<br>✓ Communication skills and general knowledge
HOLST, 2015	To examine how fever during pregnancy is associated with motor development in the child.	Denmark	Cohort	1996-2002	Children	44 256	7 years	Developmental Coordination Disorder Questionnaire	Fever during pregnancy	✓ Setting unsupported<br>✓ Walking unassisted<br>✓ Developmental coordination disorder
Trumpff, 2015	To investigate the effect of MID during late pregnancy, assessed by the thyroid-stimulating hormone (TSH) concentration at neonatal screening, on the cognitive development of preschool children	Belgium	Cohort	2008, 2009, 2010	Children age 4-6 years	315	4-6 years	Wechsler Preschool and Primary Scale of Intelligence-Fourth Edition	High household income<br>High household income<br>High household income<br>Maternal education<br>Maternal education<br>Maternal education	✓ Full scale IQ<br>✓ Verbal IQ<br>✓ Performance IQ
Ibanez, 2015	To investigate the relation between antenatal maternal depression, anxiety and children's early cognitive development among 1380 two-year-old children and 1227 three-year-old children.	Canada	Cohort	2003-2006	Children aged 2-3 years	1227	2-3 years	Communicative Development Inventory at 2 years of age and overall development with the Ages and Stages Questionnaire at 3 years of age	Antenatal maternal Depression and anxiety: Anxious, non-depressive<br>Antenatal maternal Depression and anxiety: Depressive, non-anxious<br>Antenatal maternal Depression and anxiety: Depressive and anxious	✓ CDI< threshold<br>✓ ASQ < threshold
Lertxundi, 2015	To assess whether prenatal exposure to fine particulate matter (PM2.5), nitrogen dioxide (NO2) and benzene were associated with impaired development in infants during their second year of life	Spain	Cohort	2006-2008	Mother-child dyads	438	0.5 year	Mental scale of the BSID	PM2.5 exposure during pregnancy<br>NO2 exposure during pregnancy<br>Benzene exposure during pregnancy	✓ Mental scale score<br>✓ Motor scale score

Liu, 2015	To determine the contribution of maternal, fetal and neonatal health to developmental status at ages 4–7 years	China	Cohort	2009-2010	Mother-child dyads	19 187	4-7 years	Denver Developmental Screening Test-II	Maternal age: 25-30 as compared to 20-25<br>Family income: annual > 15000 as compared to < 6000<br>Maternal education: High school and above as compared to elementary or less	✓ Developmental delay
Luo, 2015		China	Cohort	2013-2013	Mother-child dyads	1808	0.5-1 year	Bayley Scales of Infant Development	Hb concentration	✓ MDI score<br>✓ PDI score
Smithers, 2015	To examine the risk of poor child development according to week of gestation at birth, among children born ≥37 weeks gestation	Australia	Cohort	2009	Children at school entry	12 601	5 years	EDI_A	Gestational age: 37 weeks as compared to 40<br>Gestational age: 38 weeks as compared to 40<br>Gestational age: 39 weeks as compared to 40<br>Gestational age: 41 weeks as compared to 40<br>Gestational age: 42-45 weeks as compared to 40	✓ DV1<br>✓ Physical health and wellbeing<br>✓ Social competence<br>✓ Emotional maturity<br>✓ Language and cognitive skills<br>✓ Communication skills and general knowledge
Syrengelas, 2014	To investigate gross motor development in Greek infants establish AIMS percentile curves and examine the possible association of AIMS scores with socioeconomic parameters	Greek	Cross-sectional study	2008-2013	Infants	1068	2 year	Alberta Infant Motor Scale (AIMS)	Maternal education	✓ Gross motor score
Smithers, 2014	To examine whether anaemia of pregnancy is associated with adverse perinatal outcomes and with children's developmental vulnerability	Australia	Cohort	2009	Children at school entry	13 654	5 years	EDI_A	Anemia during pregnancy	✓ DV1<br>✓ Physical health and wellbeing<br>✓ Social competence<br>✓ Emotional maturity<br>✓ Language and cognitive skills<br>✓ Communication skills and general knowledge
Saleem, 2014	To examine how maternal and paternal pregnancy wantedness and couple concordance regarding pregnancy wantedness predict children's socioemotional development in kindergarten	USA	Cohort	2006-2007	Children	4650	5 years	Preschool and Kindergarten Behavior Scales – Second Edition	Mother pregnancy wantedness: unwanted	✓ Social-emotional development
Kim, 2014	To investigate the association between prenatal exposure to particulates of less than ten µm in diameter (PM10) and nitrogen dioxide (NO2) and neurodevelopment in children during the first 24 months of their lives.	South Korea	Prospective Cohort	2006	Mother-child dyads	520	0.6-2 years	K-BSID-II at 6, 12 and 24 months of age	Prenatal exposure to PM10<br>Prenatal exposure to NO2	✓ MDI<br>✓ PDI

Huang, 2014	To examine the association between maternal prepregnancy weight and child neurodevelopment and the effect of gestational weight gain	USA	Cohort	1959–76	Children	30212	7 years	Wechsler Intelligence Scales	Prepregnancy BMI: underweight<br>Prepregnancy BMI: overweight<br>Prepregnancy BMI: obesity	✓ Full-scale IQ<br>✓ Verbal scale IQ<br>✓ Performance IQ
Domingues, 2014	To evaluate if leisure-time physical activity (LTPA) during pregnancy could alter offspring's IQ and neurodevelopment during childhood in a Brazilian birth cohort.	Brazil	Cohort	2004	Mother-child dyads	3792	1-4 year	Battelle's Development Inventory and IQ	Leisure time Physical activity during pregnancy	✓ Battelle Score
Chen, 2013	To investigate the associations between in-utero exposure to perfluorooctanoic acid (PFOA) and perfluorooctyl sulfonate (PFOS) and early childhood neurodevelopment	Taiwan	Cohort	2004-2005	Mother-child dyads	239	2 years	Comprehensive Developmental Inventory for Infants and toddlers	Perfluorinated Compound Levels in Cord Blood: interquartile range increase	✓ Developmental delay<br>✓ Cognitive delay<br>✓ Language delay<br>✓ Gross motor delay<br>✓ Fine motor delay<br>✓ Social delay<br>✓ Self-help
Tran, 2013	To examine the effects of antenatal exposure to iron deficiency anemia (IDA) and common mental disorders (CMD) on the cognitive development of 6-month-old infants in a developing country.	Vietnam	Cohort	2009-2011	Pregnant women until 6 months partum	497	0.5 years	Bayley Scales of Infant and Toddler Development 3rd Ed, Cognitive Scale (BSID)	Antenatal persistent iron deficiency anaemia<br>Antenatal mental diseases	✓ Cognitive score
Curtin, 2013	To explore the potential of EDI as an indicator of early development in Ireland.	Ireland	Cross-sectional study	2003	Children at school entry	1243	5 years	EDI	Male<br>English as a second language (ESL)<br>Low birth weight<br>No preschool<br>Mother education primary or less than University<br>Screen time<br>Story telling never as compared to every day	✓ DV
Ozkan, 2012	To investigate the biological and socioeconomic factors associated with developmental attainment in socioeconomically disadvantaged children	Turkey	Cohort	2010	Children	692	0.6-5 years	Denver II	Matrnal education: illitrate<br>Paternal education: illitrate<br>Household income: below poverty level	✓ Developmental delay
Jedrychowski, 2012	To assess the effect of exclusive breastfeeding on the neurodevelopment of children over a seven-year follow-up period and to test the hypothesis that the observed cognitive gain in breastfed children in the first years of life is a strong predictor of their cognitive development trajectory, which may be continued in later life	Poland	Cohort	2001-2004	Children at school entry	468	7 years	Bayley Mental Scales of Infant Development – second edition (BSID-II) and Test of Nonverbal Intelligence (TONI-3)	Exclusive breastfeeding: <= 3months<br>Exclusive breastfeeding: 4-6months<br>Exclusive breastfeeding: >6months	✓ Cognitive score

Hinkle, 2012	To examine the relationship between maternal prepregnancy BMI status and standardized measures of mental and motor development among US children at 2 years of age.	USA	Cohort	2001	Mother and child with two years of age	6850	2 years	Bayley Scales of Infant Development-II	Maternal prepregnancy BMI: underweight as compared with Normal weight<br>Maternal prepregnancy BMI: overweight as compared with Normal weight<br>Maternal prepregnancy BMI: Obese class I as compared with Normal weight<br>Maternal prepregnancy BMI: Obese class II and III as compared with Normal weight	✓ MDI<br>✓ PDI
Quevedo, 2012	To determine which factors related to maternal depression during the first year of a child's life affect the child's language development process	Brazil	Cohort	2007-2008	Mother-child dyads	296	1 year	Bayley Scales of Infant Development III	Maternal depression	✓ Language development
Vrijheid, 2012	To examine the relationship between indoor gas cooking during pregnancy and infant neurodevelopment	Spain	Cohort	2004-2008	Mother child dyads	1887	1-2 years	Bayley Scales of Infant Development	Gas Cooking	✓ Mental development score
Brinkman, 2012	To explore population-level patterns of child development across Australian jurisdictions, considering socioeconomic and demographic characteristics	Australia	Census	2009	Children at school entry	233 960	5 years	EDI_A	Jurisdiction in male<br>Jurisdiction in female<br>Aboriginal and Torres Strait Islander: male<br>Aboriginal and Torres Strait Islander: female<br>English second language: male<br>English second language: female<br>Socioeconomic Advantage and Disadvantage Index (SEIFA IRSAD): male<br>Socioeconomic Advantage and Disadvantage Index (SEIFA IRSAD): female	✓ DV1
Wehby, 2011	To evaluate the effects of maternal smoking during pregnancy on child neurodevelopment between 3 and 24 months of age and interactions with socioeconomic status (SES)	Brazil, Argentina and Chile	Cohort	2005-2006	Children	1,584	0.5-2 years	Bayley Infant Neurodevelopmental Screener (BINS)	Smoking during pregnancy	✓ Developmental delay
Kerstjens, 2011	To determine the prevalence and nature of developmental delay at preschool age in infants born moderately preterm compared with those born full-term and early preterm	Netherlands	Prospective Cohort	2002-2003	Children at school entry	2758	Four years	Ages and Stages Questionnaire (ASQ)	Early preterm<br>Moderate preterm	✓ Total score<br>✓ Fine motor<br>✓ Gross motor<br>✓ Communication

										✓ Problem-solving<br>✓ Personal Social
Keim, 2011	To examine the associations between maternal psychological health (trait anxiety, perceived stress, depressive symptoms) during pregnancy or postpartum and infant visual, language, motor, and overall cognitive development	USA	Cohort	2001–2006	Mother-child dyads	358	One year	Mullen Scales of Early Learning	Trait anxiety score<br>Depressive symptoms: occurred <20 weeks pregnancy<br>Depressive symptoms: occurred 24-29 weeks pregnancy<br>Depressive symptoms: postpartum<br>Cohen's Perceived Stress Scale score	✓ Composite/global cognitive score<br>✓ Gross motor<br>✓ Fine motor<br>✓ Receptive language<br>✓ Expressive language
Horton, 2011		USA	Cohort	1998-2006	Mother and children at the age of 36 months	725	3 years	Bayley Scales of Infant Development, second edition	Piperonyl Butoxide in Personal	✓ Delayed Mental Development
Flick, 2011	To investigate the association between exposure to nitrogen dioxide (NO2) as a marker of traffic-related air pollution and cognitive development in children.	USA	Cohort	1976-1982	Mother-child dyads	8548	5 years	Self-developed tool	Anaesthesia and or surgery: multiple exposure	✓ Learning disability
Sansavini, 2011		Italy	Cohort	2003-2006	Children	88	2 years	Griffiths Mental Development Scales	Gestational age	✓ Developmental quotient (DQ) scores
Saha, 2010	To investigate the association of HHFS with subsequent language development of children at 18 months of age in rural Bangladesh	Bangladesh	Cohort	2002–2003	Mother-child dyads	1439	1.5 years	Bengali Adaptation of MacArthur's Communicative Development Inventory	Household food security	✓ Language comprehension<br>✓ Language expression
Freire, 2010	To investigate the association between exposure to nitrogen dioxide (NO2) as a marker of traffic-related air pollution and cognitive development in children.	Spain	Cohort	2000-2006	Mother-child dyads	210	4 years	McCarthy Scales of Children's Abilities (MSCA)	No2 exposure:15.40 - 24.75<br>No2 exposure:> 24.75	✓ Child cognitive score
De Moura, 2010	To identify risk factors for suspected developmental delay (SDD) at age 2 years among all children born in the city of Pelotas, Brazil, in 2004	Brazil	Cohort	2004	Mother-child dyads	3869	2 years	Battelle Screening Developmental Inventory (BSDI)	Male<br>Maternal schooling<br>Economic class<br>Smoking<br>Birth spacing<br>Preterm birth<br>APGAR score >6<br>Z-score length for age<br>Hospital admission<br>Presence of child book<br>Telling a story to the child	✓ Positive Battelle Screening Developmental Inventory (BSDI)
Abubakar, 2010	To investigate markers of risk status that can be easily monitored in resource-limited	Kenya	Cohort	2010	Children aged between 2-10 months	85	0.5-2 years	Local toll adapted from Griffiths Mental Developmental Scale for	Stunting<br>Underweight<br>Gravidity	✓ Developmental delay

	settings for the identification of children in need of early developmental intervention							Infants (Griffiths, 1954) and Vineland Adaptive Behaviour Scale (Sparrow, Balla, & Cicchetti, 1984)	Child health<br>Maternal schooling	
Mensah, 2010	To investigate the interplay between the mental health of mothers and fathers and family socioeconomic resources and the impact on children's cognitive and social development	England	Cohort	2005-2006	Family	4781	4-5 years	Foundation Stage Profile (FSP)	Parental mental health is measured using the Kessler scale: mother	✓ Communication, language and literacy<br>✓ Personal, social and emotional development<br>✓
Halpern, 2009	To assess developmental delay among children at one year	Brazil	Cohort	1993-2004	Mother-child dyads	3,907	1 year	Denver II screening instrument	Family income<br>Birth Weight	✓ Developmental delay
Tofail, 2009	We assessed the effects of in-utero arsenic exposure during pregnancy on infants' problem-solving ability and motor development	Bangladesh	Cohort	2002-2003	Pregnant mother and children until age 7 months	1799	1 year	problem-solving tests (PSTs), the motor scale of the Bayley Scales of Infant Development–II	Mothers' urinary arsenic (mean of weeks 8 and 30, μg/L)	✓ PDI
Deave, 2008	To examine the associations between maternal depressive symptoms during pregnancy and child development at 18 months of age	England	Prospective Cohort	1991-1992	Mother-child dyads	9244	1.5 years	Modified Denver Developmental Screening Test (modified DDST)	Postpartum depression	✓ Developmental delay
Eickmann, 2007	To investigate the association between breastfeeding and mental and motor development at age 12 months, controlling for comprehensive measures of the child's socioeconomic, maternal and environmental background and nutritional status.	Brazil	Cohort	2001	Mother-child dyads	205	1 year	Bayley Scales of Infant Development II	Breastfeeding	✓ MDI<br>✓ PDI
Julvez, 2007		Menorca Island	Cohort	1997	Parent and children	420	4 years	McCarthy Scales of Children's Abilities (MCSA)	Maternal smoking: during pregnancy<br>Maternal smoking post pregnancy only<br>Father smoking	✓ Global cognitive score
Slykerman, 2007	To determine which demographic, maternal, obstetric and postnatal variables were associated with the achievement of developmental milestones at the age of 12 months in term infants	New Zealand	Cross-sectional study	1996-1997	Mother-child pair	2182	1 year	Denver Pre-screening Developmental Questionnaire	Maternal smoking during pregnancy<br>Maternal smoking after birth within the first year<br>Low level of satisfaction with parenting	✓ Developmental delay
Perera, 2006	To evaluate the effects of prenatal exposure to airborne PAHs, estimated by personal air sampling of the mother during pregnancy, on mental and psychomotor development of children through 36 months of age, controlling for physical, biological, and	USA	Cohort	2003	Children	183	3 years	Bayley Scales of Infant Development–Revised (BSID-II)	Prenatal Exposure to Airborne Polycyclic Aromatic Hydrocarbons	✓ MDI<br>✓ Cognitive developmental delay

	psychosocial determinants of these outcomes									
Ramchandani, 2005	to assess the effect of postnatal depression on child development	UK	Cohort	1991-1992	Mother-child dyads	10 024	3.5 years	Rutter revised preschool scales	Maternal depression<br>Paternal depression	✓ Emotional developmental delay<br>✓ Prosocial<br>✓ Conduct<br>✓ Hyperactivity<br>✓ Total delay
NELSON, 2004		USA	Cohort	1994-1996	Children aged 2-4 years	143	2-4 years	Bayley Mental (MDI) and Motor (PDI) Development indices at two years, and the Wechsler Preschool and Primary Scales of Intelligence (WPPSI) and the Peabody Developmental Motor Scales (PDMS)	Iron deficiency Anaemia<br>Cociane exposure	✓ MDI<br>✓ PDI<br>✓ Full scale IQ<br>✓ Verbal IQ<br>✓ Performance ID<br>✓ Gross motor<br>✓ Fine motor
Dean,2002	To investigate the frequency of neonatal and later childhood morbidity in children exposed to antiepileptic drugs in utero	Scotland	Cohort	1976-2000	Mother-child dyads	293	1-2 years	Developmental checklist	Antiepileptic drug during pregnancy	✓ Developmental delay

Table S3. Studies reported the association between adverse early childhood developmental outcomes and various perinatal and early childhood risk factors.

Socioecological levels	*Risk factors*		*# studies*	*Association with adverse developmental outcomes*		*Citation*
				Positive	*Inverse*	
Individual level factors (n=94)	*Demographic characteristics (n=11)*	*Sex (male)*	*7*	*All positive*		1-5
		Age at assessment	*2*		*Inverse*	4 6
		English as a second language	*2*	*Positive*		3 7
	Birth outcomes related factors (n=31)	*APGAR score*	*3*		*Inverse*	8-10
		Low birth weight	*8*	*Positive*		3 5 8 11-14
		Gestational Age (preterm or post-term)	*18*	*16 Positive*		15, 5 8 15-25
		In Vitro, Fertilization-conceived children	*1*	*No significant association*		26
		Elective birth at 39 weeks	*1*	*No significant association*		27
	Child medical history (n=20)	*Childhood infectious and non-infectious diseases*	*2*	*Positive*		28 29
		Hospitalisation	*4*	*Positive*		5 8 30 31
		Chronic illness	*1*	*Positive*		32
		Child anemia	*2*	*Positive*		33
		Unaddressed dental need	*1*	*Positive*		34
		Hearing loss	*1*	*Positive*	`	35
		Childhood cancer	*1*	*Positive*		36
		Plagiocephaly	*1*	*Positive*		37
		Surgery and anaesthesia exposure	*4*	*Positive*		38-41
		HIV exposure	*2*	*Positive*		42 43
		Exposure to ART vs ZDV	*1*	*No significant association*		44
	Childhood nutritional status (n=18)	*Hb concentration*	*1*		*Inverse*	45
		Underweight	*5*	*Positive*		29, 8 46-48
		Overweight	*1*	*No significant association*		47
		Obesity	*1*	*Positive*		47

		BMI z score	*1*		*Inverse*	*49*
		Stunting	*3*	*Positive*		*29 48 50*
		Animal-source food consumption	*1*		*Inverse*	*51*
		Inadequate calorie intake	*1*	*Positive*		*52*
		Breastfeeding	*4*		*Inverse*	*14 53-55*
	Lifestyle-related Factors (n=14)	*Presence of childhood books and storytelling*	*3*		*Inverse*	*3 4 8*
		Child punishment	*1*	*Positive*		*4*
		Iron supplementation and deworming	*1*		*Inverse*	*56*
		Excessive Screen time	*7*	*Positive*		*1 3 6 57-60*
		Physical inactivity	*1*	*Positive*		*1*
		Inadequate sleep	*1*	*Positive*		*1*
Interpersonal factors (n=130)	*Sociodemographic factors (n=29)*	*Maternal age*	*5*	*U-shaped*		*5 61-66*
		Maternal education	*11*		*Inverse*	*3 4 8 11 29 52 61 63 65 67-69*
		Economic status and household income level	*11*		*Inverse*	*1 2 5 6 8 11 12 63 66 67 69 70*
		Marital status (Consanguineous marriage)	*1*	*Positive*		*61*
		Number of siblings	*1*	*Positive*		*5*
	Mental health-related factors (n=27)	*Maternal mental health issues*	*21*	*Positive*		*5 22 28 70-91*
		Maternal IQ	*1*		*Inverse*	*91*
		Prenatal exposure to mental health drugs	*5*	*Positive*		*92-96*
	Maternal health and pregnancy-related factors (n=37)	*Maternal infectious and non-infectious diseases*	*1*	*Positive*		*28*
		Placental inflammatory pathology	*1*	*Positive*		*97*
		Maternal iron deficiency anaemia	*4*	*Positives*		*98 88 99*
		Maternal overweight/obesity	*1*	*Positive*		*98 100*
		Gestational DM	*2*	*Positive*		*101 102*
		Gestational hypertension	*1*	*Positive*		*102*
		Preeclampsia	*2*	*Positive*		*103 104*

		Maternal Hospitalisation	*2*	*Positive*		5
		Maternal fever during pregnancy	*1*	*Positive*		105
		Maternal sleep apnea	*1*	*Positive*		106
		Maternal multiple sclerosis	*1*		*Inverse*	107
		Gestation weight gain (GWG)	*1*	*U shaped*		108
		Maternal pre-pregnancy BMI	*4*	*U shaped*		109-112
		Unintended pregnancy	*2*	*Positive*		113 114
		Parity	*1*	*Positive*		115
		Gravidity	*1*	*Positive*		29
		Interbirth interval	*3*	*Positive*		8 116 117
		Type of delivery C/s	*1*	*Positive*		61
		Maternal smoking	*6*	*Positive*		8 118-121
		Maternal alcohol use	*1*	*Positive*		122
	Nutrition and Lifestyle (n=12)	*Maternal conviction*	*1*	*Positive*		123
		Caregiver/mother interaction or stimulation activities	*1*		*Inverse*	124
		Knowledge of dietary diversity	*1*		*Inverse*	124
		Household food security	*1*		*Inverse*	125
		Maternal resources for care, well-nourished and support in chores	*1*		*Inverse*	126
		Sufficient Maternal vitamin B12 level during pregnancy	*1*		*Inverse*	127
		Leisure time physical activity	*1*		*Inverse*	128
		Domestic violence	*2*	*Positive*		129 130
		Intimate partner violence	*3*	*Positive*		68 80 131
	Prenatal Exposure to Substances and Environmental Factors (n=15)	*Prenatal exposure to piperonyl butoxide*	*1*	*Positive*		132
		Maternal urinary arsenic	*1*	*No significant association*		133
		Prenatal Exposure to Airborne Polycyclic Aromatic Hydrocarbons	*1*	*Positive*		134

		Corticosteroid: dexamethasone exposure during pregnancy	*1*	*Positive*		135
		Prenatal cadmium exposure	*1*	*No significant association*		6
		Prenatal Cocaine Exposure	*2*	*Positive*		100 136
		Prenatal exposure to insecticides	*1*	*Positive*		137 138
		Prenatal Mercury and perfluoroalkyl acid isomers	*1*	*Positive*		139
		Perfluorinated Compound Levels in Cord Blood	*1*	*Positive*		140
		Postnatal exposure to phthalates	*1*	*Positive*		141
		Prenatal Mancozeb Exposure, Excess Manganese	*1*	*Positive*		142
		Prenatal and childhood chlorpyrifos exposure	*1*	*Positive*		143
		Prenatal exposure to Environmental tobacco smoke	*2*	*Positive*		144 145
	Paternal factors (n=9)	*Paternal conviction*	*1*	*Positive*		123
		Paternal mental illness	*2*	*Positive*		79 83
		Paternal multiple sclerosis	*1*	*No significant association*		107
		Father smoking	*1*	*Positive*		119
		Paternal education	*2*		*Inverse*	52 67
		Parental alcohol use	*1*	*Positive*		122
		Father's pregnancy unwantedness: unwanted	*1*	*Positive*		114
	Parenting behaviours and home environment (n=11)	*Parenting behaviour: parental encouragement*	*2*		*Inverse*	146 147
		Poor child stimulation	*1*	*Positive*		52
		Parental engagement	*1*		*Inverse*	6
		Low level of satisfaction with parenting	*1*	*Positive*		120

		Home environment: physical space, variety of stimulation, fine motor and gross motor toys	*2*		*Inverse*	[4 148]
		Psychosocial and environmental determinants: organisation of the environment, play material, stimulation	*1*		*Inverse*	[149]
		Indoor air pollution: cooking fuels	*3*	*Positive*		[150-152]
Community/organization-level factors (n=36)	*Community compositions (n=6)*	*Aboriginality*	*3*	*Positive*		[20 153 154]
		Remoteness	*1*	*Positive*		[5]
		Socioeconomic index for areas	*2*	*Positive*		[5 7]
	Educational institutions (n=4)	*Preschool attendance*	*1*		*Inverse*	[3]
		Early education and care attendance	*3*		*Inverse*	[4 155 156]
	Environmental factors (n=26)	*Proximity to the school ground*	*1*		*Inverse*	[153]
		Proximity to a major road	*2*	*1 Positive*		[157]
		Proximity to the park	*1*		*Inverse*	[158]
		Air pollution (PM2.5, PM10, NO2, Benzene, SO2)	*20*	*Positive*		[159-169]
		Born season	*1*	*No significant association*		[162 170]
		Traffic load	*1*	*Positive*		[164]
Societal, policy or program-related factors (n=7)	*Policy/ program (n=7)*	*Armed conflict*	*1*	*Positive*		[171]
		Human Development Index	*1*		*Inverse*	[172]
		Jurisdiction	*2*	*Positive*	*Inverse*	[7 173]
		Health and education services before five years	*1*		*Inverse*	[174]
		Family First Home Visiting program	*1*	*No significant association*		[175]
		Healthy baby prenatal benefits	*1*	*No significant association*		[176]

N.B.: A positive association indicates that the factor increases the risk of adverse early childhood developmental outcomes. An inverse association signifies that the factor reduces this risk. No significant association means that the confidence interval for the association includes a null value, indicating no clear effect. In the table, 'n' represents the number of studies in each category.

References

1. Kerai S, Almas A, Guhn M, et al. Screen time and developmental health: results from an early childhood study in Canada. *BMC public health* 2022;22:310. doi: doi:
2. Webb S, Duku E, Brownell M, et al. Sex differences in the socioeconomic gradient of children's early development. *SSM-POPULATION HEALTH* 2020;10 doi: doi:10.1016/j.ssmph.2019.100512
3. Curtin M, Madden J, Staines A, et al. Determinants of vulnerability in early childhood development in Ireland: a cross-sectional study. *BMJ open* 2013;3(5):e002387.
4. Hasan MN, Babu MR, Chowdhury MAB, et al. Early childhood developmental status and its associated factors in Bangladesh: a comparison of two consecutive nationally representative surveys. *BMC public health* 2023;23:687. doi: doi:
5. Strobel NA, Richardson A, Shepherd CCJ, et al. Modelling factors for aboriginal and Torres strait islander child neurodevelopment outcomes: a latent class analysis. *Paediatric and Perinatal Epidemiology* 2020;34(1):48-59. doi: doi:http://dx.doi.org/10.1111/ppe.12616
6. Ma C, Iwai-Shimada M, Nakayama SF, et al. Association of prenatal exposure to cadmium with neurodevelopment in children at 2 years of age: The Japan Environment and Children's Study. *Environment international* 2021;156:106762-62. doi: 10.1016/j.envint.2021.106762
7. Brinkman SA, Gialamas A, Rahman A, et al. Jurisdictional, socioeconomic and gender inequalities in child health and development: analysis of a national census of 5-year-olds in Australia. *BMJ open* 2012;2(5):e001075.
8. De Moura DR, Costa JC, Santos IS, et al. Risk factors for suspected developmental delay at age 2 years in a Brazilian birth cohort. *Paediatric and Perinatal Epidemiology* 2010;24:211-21. doi: doi:
9. Razaz N, Boyce WT, Brownell M, et al. Five-minute Apgar score as a marker for developmental vulnerability at 5 years of age. *Archives of Disease in Childhood-Fetal and Neonatal Edition* 2016;101(2):F114-F20.
10. Razaz N, Cnattingius S, Persson M, et al. One-minute and five-minute Apgar scores and child developmental health at 5 years of age: a population-based cohort study in British Columbia, Canada. *BMJ open* 2019;9(5):e027655.
11. Sukanya G, Prabha S, Samsuzzaman M, et al. Developmental delay among children under two years of age in slums of Burdwan municipality: a cross.sectional study. *Journal of Family Medicine and Primary Care* 2021;10(5):1945-49. doi: doi:http://dx.doi.org/10.4103/jfmpc.jfmpc_1926_20
12. Halpern R, Barros AJ, Matijasevich A, et al. Developmental status at age 12 months according to birth weight and family income: a comparison of two Brazilian birth cohorts. *Cad Saude Publica* 2008;24:S444-50. doi: doi:10.1590/s0102-311x2008001500010
13. Rocha HAL, Sudfeld CR, Leite AJM, et al. Maternal and neonatal factors associated with child development in Ceara, Brazil: a population-based study. *BMC Pediatrics* 2021;21(163) doi: doi:http://dx.doi.org/10.1186/s12887-021-02623-1
14. Wang P, Hao M, Han W, et al. Factors associated with nutritional status and motor development among young children. *Nursing & Health Sciences* 2019;21(3):323-29. doi: doi:
15. Smithers LG, Searle AK, Chittleborough CR, et al. A whole-of-population study of term and post-term gestational age at birth and children's development. *BJOG-AN INTERNATIONAL JOURNAL OF OBSTETRICS AND GYNAECOLOGY* 2015;122(10):1303-11. doi: doi:10.1111/1471-0528.13324

16. Crockett L, Ruth C, Heaman M, et al. Education outcomes of children born late preterm: a retrospective whole-population cohort study. *Maternal and Child Health Journal* 2022;26(5):1126-41.
17. Ballantyne M, Benzies KM, McDonald S, et al. Risk of developmental delay: Comparison of late preterm and full term Canadian infants at age 12 months. *EARLY HUMAN DEVELOPMENT* 2016;101:27-32. doi: doi:10.1016/j.earlhumdev.2016.04.004
18. Dhamrait GK, Christian H, O'Donnell M, et al. Gestational age and child development at school entry. *SCIENTIFIC REPORTS* 2021;11(1) doi: doi:10.1038/s41598-021-93701-y
19. Gleason JL, Gilman SE, Sundaram R, et al. Gestational age at term delivery and children's neurocognitive development. *INTERNATIONAL JOURNAL OF EPIDEMIOLOGY* 2021;50(6):1814-23. doi: doi:10.1093/ije/dyab134
20. Hanly M, Falster K, Chambers G, et al. Gestational Age and Child Development at Age Five in a Population-Based Cohort of Australian Aboriginal and Non-Aboriginal Children. *Paediatr Perinat Epidemiol* 2018;32(1):114-25. doi: doi:10.1111/ppe.12426
21. Hua J, Barnett AL, Lin Y, et al. Association of Gestational Age at Birth With Subsequent Neurodevelopment in Early Childhood: A National Retrospective Cohort Study in China. *FRONTIERS IN PEDIATRICS* 2022;10 doi: doi:10.3389/fped.2022.860192
22. Junge C, Garthus-Niegel S, Slinning K, et al. The Impact of Perinatal Depression on Children's Social-Emotional Development: A Longitudinal Study. *MATERNAL AND CHILD HEALTH JOURNAL* 2017;21(3):607-15. doi: doi:10.1007/s10995-016-2146-2
23. Kerstjens JM, de Winter AF, Bocca-Tjeertes IF, et al. Developmental delay in moderately preterm-born children at school entry. *J Pediatr* 2011;159(1):92-8. doi: doi:10.1016/j.jpeds.2010.12.041
24. Richards JL, Drews-Botsch C, Sales JM, et al. Describing the Shape of the Relationship Between Gestational Age at Birth and Cognitive Development in a Nationally Representative U.S. Birth Cohort. *Paediatr Perinat Epidemiol* 2016;30(6):571-82. doi: doi:10.1111/ppe.12319
25. Sansavini A, Savini S, Guarini A, et al. The effect of gestational age on developmental outcomes: a longitudinal study in the first 2 years of life. *Child: care, health and development* 2011;37:26-36. doi: doi:
26. Kennedy AL, Vollenhoven BJ, Hiscock RJ, et al. School-age outcomes among IVF-conceived children: A population-wide cohort study. *PLoS Med* 2023;20(1):e1004148. doi: doi:10.1371/journal.pmed.1004148
27. Lindquist A, Hastie R, Kennedy A, et al. Developmental outcomes for children after elective birth at 39 weeks' gestation. *JAMA pediatrics* 2022;176(7):654-63.
28. Green MJ, Kariuki M, Dean K, et al. Childhood developmental vulnerabilities associated with early life exposure to infectious and noninfectious diseases and maternal mental illness. *Journal of child psychology and psychiatry, and allied disciplines* 2018;59:801-10. doi: doi:
29. Abubakar A, Holding P, Van de Vijver FJR, et al. Children at risk for developmental delay can be recognised by stunting, being underweight, ill health, little maternal schooling or high gravidity. *JOURNAL OF CHILD PSYCHOLOGY AND PSYCHIATRY* 2010;51(6):652-59. doi: doi:10.1111/j.1469-7610.2009.02193.x
30. Kariuki M, Raudino A, Green MJ, et al. Hospital admission for infection during early childhood influences developmental vulnerabilities at age 5 years. *Journal of Paediatrics and Child Health* 2016;52(9):882-88. doi: doi:http://dx.doi.org/10.1111/jpc.13239
31. Fardell JE, Hu N, Wakefield CE, et al. Impact of Hospitalizations due to Chronic Health Conditions on Early Child Development. *JOURNAL OF PEDIATRIC PSYCHOLOGY* 2023;48(10):799-811. doi: doi:10.1093/jpepsy/jsad025

32. Bell MF, Bayliss DM, Glauert R, et al. Chronic illness and developmental vulnerability at school entry. *Pediatrics* 2016;137 doi: doi:
33. Leonard D, Buettner P, Thompson F, et al. Early childhood anaemia more than doubles the risk of developmental vulnerability at school‐age among Aboriginal and Torres Strait Islander children of remote Far North Queensland: Findings of a retrospective cohort study. *Nutrition & Dietetics* 2020;77(3):298-309. doi: doi:10.1111/1747-0080.12602
34. Janus M, Reid-Westoby C, Lee C, et al. Association between severe unaddressed dental needs and developmental health at school entry in Canada: a cross-sectional study. *BMC pediatrics* 2019;19:1-9.
35. Simpson A, Šarkić B, Enticott JC, et al. Developmental vulnerability of Australian school-entry children with hearing loss. *Australian Journal of Primary Health* 2020;26(1):70-75.
36. Morris JN, Roder D, Turnbull D, et al. The impact of cancer on early childhood development: A linked data study. *Journal of Pediatric Psychology* 2021;46(1):49-58.
37. Rohde JF, Goyal NK, Slovin SR, et al. Association of positional plagiocephaly and developmental delay within a primary care network. *Journal of Developmental and Behavioral Pediatrics* 2021;42(2):128-34. doi: doi:
38. Graham MR, Brownell M, Chateau DG, et al. Neurodevelopmental Assessment in Kindergarten in Children Exposed to General Anesthesia before the Age of 4 Years. *Anesthesiology* 2016;125:667-77. doi: doi:
39. O'Leary JD, Janus M, Duku E, et al. Influence of Surgical Procedures and General Anesthesia on Child Development Before Primary School Entry Among Matched Sibling Pairs. *JAMA Pediatr* 2019;173(1):29-36. doi: doi:10.1001/jamapediatrics.2018.3662
40. O'Leary JD, Janus M, Duku E, et al. A population-based study evaluating the association between surgery in early life and child development at primary school entry. *Anesthesiology* 2016;125(2):272-79.
41. Flick RP, Katusic SK, Colligan RC, et al. Cognitive and behavioral outcomes after early exposure to anesthesia and surgery. *Pediatrics* 2011;128:e1053-e61. doi: doi:
42. Roux SMl, Donald KA, Brittain K, et al. Neurodevelopment of breastfed HIV-exposed uninfected and HIV-unexposed children in South Africa. *AIDS* 2018;32(13):1781-91. doi: doi:http://dx.doi.org/10.1097/QAD.0000000000001872
43. Wu J, Li J, Li Y, et al. Neurodevelopmental outcomes in young children born to HIV-positive mothers in rural Yunnan, China. *Pediatrics International* 2018;60(7):618-25. doi: doi:
44. Chaudhury S, Mayondi GK, Williams PL, et al. In-utero exposure to antiretrovirals and neurodevelopment among HIV-exposed-uninfected children in Botswana. *AIDS* 2018;32(9):1173-83. doi: doi:http://dx.doi.org/10.1097/QAD.0000000000001790
45. Luo R, Shi Y, Zhou H, et al. Micronutrient deficiencies and developmental delays among infants: evidence from a cross-sectional survey in rural China. *BMJ Open* 2015;5(10):e008400. doi: doi:10.1136/bmjopen-2015-008400
46. O'Neill SM, Hannon G, Khashan AS, et al. Thin-for-gestational age infants are at increased risk of neurodevelopmental delay at 2 years. *Arch Dis Child Fetal Neonatal Ed* 2017;102(3):F197-f202. doi: doi:10.1136/archdischild-2016-310791
47. Pearce A, Scalzi D, Lynch J, et al. Do thin, overweight and obese children have poorer development than their healthy-weight peers at the start of school? Findings from a South Australian data linkage study. *Early childhood research quarterly* 2016;35:85-94.
48. Shrestha ML, Perry KE, Thapa B, et al. Malnutrition matters: Association of stunting and underweight with early childhood development indicators in

Nepal. *MATERNAL AND CHILD NUTRITION* 2022;18(2) doi: doi:10.1111/mcn.13321
49. Wei X, Hu J, Yang L, et al. Bidirectional association of neurodevelopment with growth: a prospective cohort study. *BMC Pediatrics* 2021;21(203) doi: doi:http://dx.doi.org/10.1186/s12887-021-02655-7
50. Allel K, Abou Jaoude G, Poupakis S, et al. Exploring the Associations between Early Childhood Development Outcomes and Ecological Country-Level Factors across Low- and Middle-Income Countries. *INTERNATIONAL JOURNAL OF ENVIRONMENTAL RESEARCH AND PUBLIC HEALTH* 2021;18(7) doi: doi:10.3390/ijerph18073340
51. Pokharel A, Webb P, Miller LC, et al. Relationship between Animal Sourced Food Consumption and Early Childhood Development Outcomes. *Nutrients* 2023;15(2):315. doi: doi:10.3390/nu15020315
52. Utami NH, ayani, Sekartini R, et al. Cognitive performance of 4 to 6-year-old children: a longitudinal study. *Paediatrica Indonesiana* 2023;63(2):65-72. doi: doi:10.14238/pi63.2.2023.65-72
53. Eickmann SH, de Lira PI, Lima Mde C, et al. Breast feeding and mental and motor development at 12 months in a low-income population in northeast Brazil. *Paediatr Perinat Epidemiol* 2007;21(2):129-37. doi: doi:10.1111/j.1365-3016.2007.00795.x
54. Jedrychowski W, Perera F, Jankowski J, et al. Effect of exclusive breastfeeding on the development of children's cognitive function in the Krakow prospective birth cohort study. *European Journal of Pediatrics* 2012;171(1):151-58. doi: doi:10.1007/s00431-011-1507-5
55. Wallenborn JT, Levine GA, Carreira dos Santos A, et al. Breastfeeding, Physical Growth, and Cognitive Development. *Pediatrics* 2021;147(5):1-10. doi: doi:10.1542/peds.2020-008029
56. Gao YQ, Wang YP, Zou SY, et al. Association of iron supplementation and deworming with early childhood development: analysis of Demographic and Health Surveys in ten low- and middle-income countries. *EUROPEAN JOURNAL OF NUTRITION* 2021;60(6):3119-30. doi: doi:10.1007/s00394-021-02493-4
57. Leao OAD, Bertoldi AD, Domingues MR, et al. Cross-sectional and prospective associations between screen time and childhood neurodevelopment in two Brazilian cohorts born 11 years apart. *CHILD CARE HEALTH AND DEVELOPMENT* 2023 doi: doi:10.1111/cch.13165
58. Rithipukdee N, Kusol K. Factors Associated with the Suspected Delay in the Language Development of Early Childhood in Southern Thailand. *Children* 2022;9(5):662-N.PAG. doi: doi:10.3390/children9050662
59. Rocha HAL, Correia LL, Leite AM, et al. Screen time and early childhood development in Ceara, Brazil: a population-based study. *BMC public health* 2021;21:2072. doi: doi:
60. Varadarajan S, Venguidesvarane AG, Ramaswamy KN, et al. Prevalence of excessive screen time and its association with developmental delay in children aged <5 years: A population-based cross-sectional study in India. *PLOS ONE* 2021;16(7) doi: doi:10.1371/journal.pone.0254102
61. Demirci A, Kartal M. The prevalence of developmental delay among children aged 3-60 months in Izmir, Turkey. *Child: care, health and development* 2016;42:213-19. doi: doi:
62. Falster K, Hanly M, Banks E, et al. Maternal age and offspring developmental vulnerability at age five: A population-based cohort study of Australian children. *PLOS MEDICINE* 2018;15(4) doi: doi:10.1371/journal.pmed.1002558
63. Liu Y, Li XN, Sun XR, et al. Prenatal and neonatal risk factors associated with children's developmental status at ages 4-7: lessons from the Jiangsu China birth defects prevention cohort. *Child: care, health and development* 2015;41:712-21. doi: doi:

64. Moreno-Giménez A, Campos-Berga L, Nowak A, et al. Impact of maternal age on infants' emotional regulation and psychomotor development. *PSYCHOLOGICAL MEDICINE* 2022;52(15):3708-19. doi: doi:10.1017/S0033291721000568
65. Syrengelas D, Kalampoki V, Kleisiouni P, et al. Gross motor development in full-term Greek infants assessed by the Alberta Infant Motor Scale: reference values and socioeconomic impact. *Early Hum Dev* 2014;90(7):353-7. doi: doi:10.1016/j.earlhumdev.2014.04.011
66. Taylor CL, Christensen D, Stafford J, et al. Associations between clusters of early life risk factors and developmental vulnerability at age 5: a retrospective cohort study using population-wide linkage of administrative data in Tasmania, Australia. *BMJ open* 2020;10(4):e033795.
67. Ozkan M, Senel S, Arslan EA, et al. The socioeconomic and biological risk factors for developmental delay in early childhood. *European Journal of Pediatrics* 2012;171(12):1815-21. doi: doi:10.1007/s00431-012-1826-1
68. Peterson CC, Riggs J, Guyon-Harris K, et al. Effects of Intimate Partner Violence and Home Environment on Child Language Development in the First 3 Years of Life. *Journal of developmental and behavioral pediatrics : JDBP* 2019;40:112-21. doi: doi:
69. Trumpff C, De Schepper J, erfaeillie J, et al. Thyroid-Stimulating Hormone (TSH) Concentration at Birth in Belgian Neonates and Cognitive Development at Preschool Age. *Nutrients* 2015;7(11):9018-32. doi: doi:10.3390/nu7115450
70. Smith TA, Kievit RA, Astle DE. Maternal mental health mediates links between socioeconomic status and child development. *CURRENT PSYCHOLOGY* 2023;42(25):21967-78. doi: doi:10.1007/s12144-022-03181-0
71. Bell MF, Glauert R, Roos LL, et al. Examining the relationship between maternal mental health-related hospital admissions and childhood developmental vulnerability at school entry in Canada and Australia. *BJPSYCH OPEN* 2023;9(1) doi: doi:10.1192/bjo.2022.642
72. Burger M, Einspieler C, Niehaus DJ, et al. Maternal mental health and infant neurodevelopment at 6 months in a low‐income South African cohort. *Infant Mental Health Journal* 2022;43(6):849-63.
73. Cornish AM, McMahon CA, Ungerer JA, et al. Postnatal depression and infant cognitive and motor development in the second postnatal year: The impact of depression chronicity and infant gender. *INFANT BEHAVIOR & DEVELOPMENT* 2005;28(4):407-17. doi: doi:10.1016/j.infbeh.2005.03.004
74. Deave T, Heron J, Evans J, et al. The impact of maternal depression in pregnancy on early child development. *BJOG-AN INTERNATIONAL JOURNAL OF OBSTETRICS AND GYNAECOLOGY* 2008;115(8):1043-51. doi: doi:10.1111/j.1471-0528.2008.01752.x
75. González G, Moraes M, Sosa C, et al. Maternal postnatal depression and its impact on child neurodevelopment: a cohort study. *REVISTA CHILENA DE PEDIATRIA-CHILE* 2017;88(3):360-65. doi: doi:10.4067/S0370-41062017000300008
76. Gül H, Gül A, Kara K. Maternal depression, anxiety, psychoticism and paranoid ideation have effects on developmental delay types of infants: A study with clinical infant-mother dyads. *ARCHIVES OF PSYCHIATRIC NURSING* 2020;34(3):184-90. doi: doi:10.1016/j.apnu.2020.04.009
77. Ibanez G, Bernard JY, Rondet C, et al. Effects of Antenatal Maternal Depression and Anxiety on Children's Early Cognitive Development: A Prospective Cohort Study. *PLOS ONE* 2015;10(8) doi: doi:10.1371/journal.pone.0135849
78. Keim SA, Daniels JL, Dole N, et al. A prospective study of maternal anxiety, perceived stress, and depressive symptoms in relation to infant cognitive development. *EARLY HUMAN DEVELOPMENT* 2011;87(5):373-80. doi: doi:10.1016/j.earlhumdev.2011.02.004
79. Mensah FK, Kiernan KE. Parents' mental health and children's cognitive and social development: families in England in the Millennium Cohort Study. *Soc Psychiatry Psychiatr Epidemiol* 2010;45(11):1023-35. doi: doi:10.1007/s00127-009-0137-y
80. Neamah HH, Sudfeld C, McCoy DC, et al. Intimate partner violence, depression, and child growth and development. *Pediatrics* 2018;142 doi: doi:

81. Polte C, Junge C, von Soest T, et al. Impact of Maternal Perinatal Anxiety on Social-Emotional Development of 2-Year-Olds, A Prospective Study of Norwegian Mothers and Their Offspring: The Impact of Perinatal Anxiety on Child Development. *MATERNAL AND CHILD HEALTH JOURNAL* 2019;23(3):386-96. doi: doi:10.1007/s10995-018-2684-x
82. Quevedo L, Silva R, Godoy R, et al. The impact of maternal post-partum depression on the language development of children at 12 months. *Child: Care, Health and Development* 2012;38(3):420-24. doi: doi:
83. Ramch, ani P, Stein A, et al. Paternal depression in the postnatal period and child development: a prospective population study. *LANCET* 2005;365(9478):2201-05. doi: doi:10.1016/S0140-6736(05)66778-5
84. Rogers AM, Youssef GJ, Teague S, et al. Association of maternal and paternal perinatal depression and anxiety with infant development: A longitudinal study. *JOURNAL OF AFFECTIVE DISORDERS* 2023;338:278-88. doi: doi:10.1016/j.jad.2023.06.020
85. Roy R, Chakraborty M, Bhattacharya K, et al. Impact of perinatal maternal depression on child development. *INDIAN JOURNAL OF PSYCHIATRY* 2022;64(3):284-88. doi: doi:10.4103/indianjpsychiatry.indianjpsychiatry_1318_20
86. Shuffrey LC, Sania A, Brito NH, et al. Association of maternal depression and anxiety with toddler social-emotional and cognitive development in South Africa: a prospective cohort study. *BMJ OPEN* 2022;12(4) doi: doi:10.1136/bmjopen-2021-058135
87. Smith-Nielsen J, Lange T, Wendelboe KI, et al. Associations Between Maternal Postpartum Depression, Infant Social Behavior With a Stranger, and Infant Cognitive Development. *INFANCY* 2019;24(4):663-70. doi: doi:10.1111/infa.12287
88. Tran TD, Biggs B-A, Tran T, et al. Impact on infants' cognitive development of antenatal exposure to iron deficiency disorder and common mental disorders. *Plos one* 2013;8(9):e74876.
89. Urizar GG, Jr., Muñoz RF. Role of Maternal Depression on Child Development: A Prospective Analysis from Pregnancy to Early Childhood. *CHILD PSYCHIATRY & HUMAN DEVELOPMENT* 2022;53(3):502-14. doi: doi:10.1007/s10578-021-01138-1
90. Wall-Wieler E, Roos LL, Gotlib IH. Maternal depression in early childhood and developmental vulnerability at school entry. *Pediatrics* 2020;146 doi: doi:
91. Zheng S, Bishop SL, Ceja T, et al. Neurodevelopmental profiles of preschool-age children in Flint, Michigan: a latent profile analysis. *Journal of Neurodevelopmental Disorders* 2021;13 doi: doi:
92. Dean JCS, Hailey H, Moore SJ, et al. Long term health and neurodevelopment in children exposed to antiepileptic drugs before birth. *JOURNAL OF MEDICAL GENETICS* 2002;39(4):251-59. doi: doi:10.1136/jmg.39.4.251
93. al M, Skurtveit S, Furu K, et al. Motor development in children prenatally exposed to selective serotonin reuptake inhibitors: a large population-based pregnancy cohort study. *Bjog* 2016;123(12):1908-17. doi: doi:10.1111/1471-0528.13582
94. Handal M, Skurtveit S, Furu K, et al. Motor development in children prenatally exposed to selective serotonin reuptake inhibitors: a large population ‐ based pregnancy cohort study. *BJOG: An International Journal of Obstetrics & Gynaecology* 2016;123(12):1908-17.
95. van der Veere CN, de Vries NKS, van Braeckel K, et al. Intra-uterine exposure to selective serotonin reuptake inhibitors (SSRIs), maternal psychopathology, and neurodevelopment at age 2.5years - Results from the prospective cohort SMOK study. *Early Hum Dev* 2020;147:105075. doi: doi:10.1016/j.earlhumdev.2020.105075
96. Singal D, Chateau D, Struck S, et al. In utero antidepressants and neurodevelopmental outcomes in kindergarteners. *Pediatrics* 2020;145(5)
97. Chen C, Lu D, Xue L, et al. Association between Placental Inflammatory Pathology and Offspring Neurodevelopment at 8 Months and 4 and 7 Years of

Age. *J Pediatr* 2020;225:132-37.e2. doi: doi:10.1016/j.jpeds.2020.05.049
98. Berglund SK, Torres-Espínola FJ, García-Valdés L, et al. The impacts of maternal iron deficiency and being overweight during pregnancy on neurodevelopment of the offspring. *Br J Nutr* 2017;118(7):533-40. doi: doi:10.1017/s0007114517002410
99. Smithers LG, Gialamas A, Scheil W, et al. Anaemia of Pregnancy, Perinatal Outcomes and Children's Developmental Vulnerability: a Whole-of-Population Study. *PAEDIATRIC AND PERINATAL EPIDEMIOLOGY* 2014;28(5):381-90. doi: doi:10.1111/ppe.12149
100. Nelson S, Lerner E, Needlman R, et al. Cocaine, anemia, and neurodevelopmental outcomes in children: A longitudinal study. *Journal of Developmental and Behavioral Pediatrics* 2004;25:1-9. doi: doi:
101. Duko B, Gebremedhin AT, Tessema GA, et al. Influence of preterm birth on the association between gestational diabetes mellitus and childhood developmental vulnerability: a causal mediation analysis. *WORLD JOURNAL OF PEDIATRICS* 2023 doi: doi:10.1007/s12519-023-00741-7
102. Ghassabian A, Sundaram R, Wylie A, et al. Maternal medical conditions during pregnancy and gross motor development up to age 24 months in the Upstate KIDS study. *Developmental Medicine and Child Neurology* 2016;58:728-34. doi: doi:
103. Duko B, Gebremedhin AT, Tessema GA, et al. The effects of pre-eclampsia on social and emotional developmental vulnerability in children at age five in Western Australia: A population data linkage study. *Journal of Affective Disorders* 2024
104. Warshafsky C, Pudwell J, Walker M, et al. Prospective assessment of neurodevelopment in children following a pregnancy complicated by severe pre-eclampsia. *BMJ OPEN* 2016;6(7) doi: doi:10.1136/bmjopen-2015-010884
105. Holst C, Jorgensen SE, Wohlfahrt J, et al. Fever during pregnancy and motor development in children: A study within the Danish National Birth Cohort. *Developmental Medicine & Child Neurology* 2015;57(8):725-32. doi: doi:
106. Bin YS, Cistulli PA, Roberts CL, et al. Childhood health and educational outcomes associated with maternal sleep apnea: a population record-linkage study. *Sleep* 2017;40(11):zsx158.
107. Razaz N, Joseph K, Boyce WT, et al. Children of chronically ill parents: Relationship between parental multiple sclerosis and childhood developmental health. *Multiple Sclerosis Journal* 2016;22(11):1452-62.
108. Motoki N, Inaba Y, Shibazaki T, et al. Insufficient maternal gestational weight gain and infant neurodevelopment at 12 months of age: the Japan Environment and Children's Study. *European Journal of Pediatrics* 2022;181(3):921-31. doi: doi:10.1007/s00431-021-04232-7
109. Hao XM, Lu JR, Yan SQ, et al. Maternal Pre-Pregnancy Body Mass Index, Gestational Weight Gain and Children's Cognitive Development: A Birth Cohort Study. *NUTRIENTS* 2022;14(21) doi: doi:10.3390/nu14214613
110. Hinkle SN, Schieve LA, Stein AD, et al. Associations between maternal prepregnancy body mass index and child neurodevelopment at 2 years of age. *Int J Obes (Lond)* 2012;36(10):1312-9. doi: doi:10.1038/ijo.2012.143
111. Huang L, Yu X, Keim S, et al. Maternal prepregnancy obesity and child neurodevelopment in the Collaborative Perinatal Project. *Int J Epidemiol* 2014;43(3):783-92. doi: doi:10.1093/ije/dyu030
112. Widen EM, Nichols AR, Kahn LG, et al. Prepregnancy obesity is associated with cognitive outcomes in boys in a low-income, multiethnic birth cohort. *BMC Pediatrics* 2019;19(507) doi: doi:http://dx.doi.org/10.1186/s12887-019-1853-4
113. Jang M, Molino AR, Ribeiro MV, et al. Maternal Pregnancy Intention and Developmental Outcomes in Brazilian Preschool-Aged Children. *Journal of developmental and behavioral pediatrics : JDBP* 2021;42:e15-e23. doi: doi:

114. Saleem HT, Surkan PJ. Parental pregnancy wantedness and child social-emotional development. *Matern Child Health J* 2014;18(4):930-8. doi: doi:10.1007/s10995-013-1320-z
115. Islam MM, Khan MN. Early childhood development and its association with maternal parity. *CHILD CARE HEALTH AND DEVELOPMENT* 2023;49(1):80-89. doi: doi:10.1111/cch.13011
116. Dhamrait GK, Taylor CL, Pereira G. Interpregnancy intervals and child development at age 5: a population data linkage study. *BMJ open* 2021;11(3):e045319.
117. Dhamrait G, O'Donnell M, Christian H, et al. Is early childhood development impeded by the birth timing of the younger sibling? *Plos one* 2022;17(5):e0268325.
118. Duko B, Gebremedhin AT, Tessema GA, et al. Average treatment effect of maternal prenatal tobacco smoking on offspring developmental vulnerability in early childhood. *ANNALS OF EPIDEMIOLOGY* 2023;78:35-43. doi: doi:10.1016/j.annepidem.2022.12.007
119. Julvez J, Ribas-Fito N, Torrent M, et al. Maternal smoking habits and cognitive development of children at age 4 years in a population-based birth cohort. *International Journal of Epidemiology* 2007;36:825-32. doi: doi:
120. Slykerman RF, Thompson JMD, Clark PM, et al. Determinants of developmental delay in infants aged 12 months. *Paediatric and Perinatal Epidemiology* 2007;21(2):121-28. doi: doi:http://dx.doi.org/10.1111/j.1365-3016.2007.00796.x
121. Wehby GL, Prater K, McCarthy AM, et al. The Impact of Maternal Smoking during Pregnancy on Early Child Neurodevelopment. *JOURNAL OF HUMAN CAPITAL* 2011;5(2):207-54. doi: doi:10.1086/660885
122. McCormack C, Hutchinson D, Burns L, et al. Maternal and partner prenatal alcohol use and infant cognitive development. *Drug Alcohol Depend* 2018;185:330-38. doi: doi:10.1016/j.drugalcdep.2017.12.038
123. Bell MF, Bayliss DM, Glauert R, et al. Using linked data to investigate developmental vulnerabilities in children of convicted parents. *Developmental psychology* 2018;54:1219-31. doi: doi:
124. Russell AL, Hentschel E, Fulcher I, et al. Caregiver parenting practices, dietary diversity knowledge, and association with early childhood development outcomes among children aged 18-29months in Zanzibar, Tanzania: a cross-sectional survey. *BMC public health* 2022;22:762. doi: doi:
125. Saha K, Tofail F, Frongillo E, et al. Household food security is associated with early childhood language development: Results from a longitudinal study in rural Bangladesh. *Child: Care, Health and Development* 2010;36(3):309-16. doi: doi:
126. Basnet S, Frongillo EA, Nguyen PH, et al. Maternal resources for care are associated with child growth and early childhood development in Bangladesh and Vietnam. *Child: Care, Health & Development* 2022;48(1):120-28. doi: doi:10.1111/cch.12911
127. Cruz-Rodríguez J, Díaz-López A, Canals-Sans J, et al. Maternal Vitamin B12 Status during Pregnancy and Early Infant Neurodevelopment: The ECLIPSES Study. *Nutrients* 2023;15(6):1529. doi: doi:10.3390/nu15061529
128. Domingues MR, Matijasevich A, Barros AJ, et al. Physical activity during pregnancy and offspring neurodevelopment and IQ in the first 4 years of life. *PLoS ONE Vol 9,(10), 2014, ArtID e110050* 2014;9(10) doi: doi:
129. Turunç G, Kisbu-Sakarya Y. Parents' Attitudes Toward Domestic Violence as a Risk Factor for Early Childhood Development: Testing an Actor-Partner Interdependence Model Using UNICEF MICS. *JOURNAL OF INTERPERSONAL VIOLENCE* 2022;37(23):NP21476-NP501. doi: doi:10.1177/08862605211058212

130. Whitten T, Green MJ, Tzoumakis S, et al. Early developmental vulnerabilities following exposure to domestic violence and abuse: Findings from an Australian population cohort record linkage study. *Journal of psychiatric research* 2022;153:223-28.
131. Jeong J, Adhia A, Bhatia A, et al. Intimate partner violence, maternal and paternal parenting, and early child development. *Pediatrics* 2020;145 doi: doi:
132. Horton MK, Rundle A, Camann DE, et al. Impact of prenatal exposure to piperonyl butoxide and permethrin on 36-month neurodevelopment. *Pediatrics* 2011;127(3):e699-706. doi: doi:10.1542/peds.2010-0133
133. Tofail F, Vahter M, Hamadani JD, et al. Effect of arsenic exposure during pregnancy on infant development at 7 months in rural Matlab, Bangladesh. *Environmental Health Perspectives* 2009;117(2):288-93. doi: doi:http://dx.doi.org/10.1289/ehp.11670
134. Perera FP, Rauh V, Whyatt RM, et al. Effect of prenatal exposure to airborne polycyclic aromatic hydrocarbons on neurodevelopment in the first 3 years of life among inner-city children. *Environmental Health Perspectives* 2006;114(8):1287-92. doi: doi:10.1289/ehp.9084
135. Tao SY, Du JB, Chi X, et al. Associations between antenatal corticosteroid exposure and neurodevelopment in infants. *AMERICAN JOURNAL OF OBSTETRICS AND GYNECOLOGY* 2022;227(5) doi: doi:10.1016/j.ajog.2022.05.060
136. Morrow CE, stra ES, Anthony JC, et al. Influence of prenatal cocaine exposure on early language development: Longitudinal findings from four months to three years of age. *Journal of Developmental and Behavioral Pediatrics* 2003;24:39-50. doi: doi:
137. Aizhen W, Yanjian W, Mahai G, et al. Association of Prenatal Exposure to Organophosphate, Pyrethroid, and Neonicotinoid Insecticides with Child Neurodevelopment at 2 Years of Age: A Prospective Cohort Study. *Environmental Health Perspectives* 2023;131(10):107011-1-11-15. doi: doi:10.1289/EHP12097
138. Wang A, Wan Y, Mahai G, et al. Association of prenatal exposure to organophosphate, pyrethroid, and neonicotinoid insecticides with child neurodevelopment at 2 years of age: a prospective cohort study. *Environmental Health Perspectives* 2023;131(10) doi: doi:http://dx.doi.org/10.1289/EHP12097
139. Reardon AJF, Hajihosseini M, Dinu I, et al. Maternal co-exposure to mercury and perfluoroalkyl acid isomers and their associations with child neurodevelopment in a Canadian birth cohort. *ENVIRONMENT INTERNATIONAL* 2023;178 doi: doi:10.1016/j.envint.2023.108087
140. Chen MH, Ha EH, Liao HF, et al. Perfluorinated compound levels in cord blood and neurodevelopment at 2 years of age. *Epidemiology* 2013;24:800-08. doi: doi:
141. Kim JH, Moon N, Ji E, et al. Effects of postnatal exposure to phthalate, bisphenol a, triclosan, parabens, and per- and poly-fluoroalkyl substances on maternal postpartum depression and infant neurodevelopment: a korean mother-infant pair cohort study. *ENVIRONMENTAL SCIENCE AND POLLUTION RESEARCH* 2023;30(42):96384-99. doi: doi:10.1007/s11356-023-29292-0
142. Mora AM, Cordoba L, Cano JC, et al. Prenatal mancozeb exposure, excess manganese, and neurodevelopment at 1 year of age in the Infants' Environmental Health (ISA) study. *Environmental Health Perspectives* 2018;126(5):EHP1955. doi: doi:http://dx.doi.org/10.1289/ehp1955
143. Guo J, Zhang J, Wu C, et al. Associations of prenatal and childhood chlorpyrifos exposure with Neurodevelopment of 3-year-old children. *Environ Pollut* 2019;251:538-46. doi: doi:10.1016/j.envpol.2019.05.040
144. He Y, Luo RF, Wang TY, et al. Prenatal Exposure to Environmental Tobacco Smoke and Early Development of Children in Rural Guizhou Province, China. *INTERNATIONAL JOURNAL OF ENVIRONMENTAL RESEARCH AND PUBLIC HEALTH* 2018;15(12) doi: doi:10.3390/ijerph15122866
145. Polanska K, Krol A, Merecz-Kot D, et al. Environmental Tobacco Smoke Exposure during Pregnancy and Child Neurodevelopment. *INTERNATIONAL*

JOURNAL OF ENVIRONMENTAL RESEARCH AND PUBLIC HEALTH 2017;14(7) doi: doi:10.3390/ijerph14070796
146. Rivero M, Vilaseca R, Cantero M-J, et al. Relations between Positive Parenting Behavior during Play and Child Language Development at Early Ages. *Children* 2023;10(3):505. doi: doi:10.3390/children10030505
147. Rocha HAL, Correia LL, Leite ÁJM, et al. Positive Parenting Behaviors and Child Development in Ceará, Brazil: A Population-Based Study. *Children* 2022;9(8):1246. doi: doi:10.3390/children9081246
148. Cao Z, Su X, Ni Y, et al. Association between the home environment and development among 3- to 11-month infants in Shanghai, China. *Child: Care, Health and Development* 2022;48(1):45-54. doi: doi:http://dx.doi.org/10.1111/cch.12902
149. Drago F, Scharf RJ, Maphula A, et al. Psychosocial and environmental determinants of child cognitive development in rural south africa and tanzania: findings from the mal-ed cohort. *BMC public health* 2020;20:505. doi: doi:
150. Grippo A, Zhu KX, Yeung EH, et al. Indoor air pollution exposure and early childhood development in the Upstate KIDS Study. *ENVIRONMENTAL RESEARCH* 2023;234 doi: doi:10.1016/j.envres.2023.116528
151. Rana J, Luna-Gutiérrez P, Haque SE, et al. Associations between household air pollution and early child development among children aged 36-59months in Bangladesh. *JOURNAL OF EPIDEMIOLOGY AND COMMUNITY HEALTH* 2022;76(7):667-76. doi: doi:10.1136/jech-2021-217554
152. Vrijheid M, Martinez D, Aguilera I, et al. Indoor air pollution from gas cooking and infant neurodevelopment. *Epidemiology* 2012;23(1):23-32. doi: doi:http://dx.doi.org/10.1097/EDE.0b013e31823a4023
153. Christian H, Ball SJ, Zubrick SR, et al. Relationship between the neighbourhood built environment and early child development. *Health & place* 2017;48:90-101.
154. Williamson A, Gibberd A, Hanly MJ, et al. Social and emotional developmental vulnerability at age five in Aboriginal and non-Aboriginal children in New South Wales: a population data linkage study. *International journal for equity in health* 2019;18:1-12.
155. Falster K, Hanly M, Edwards B, et al. Preschool attendance and developmental outcomes at age five in Indigenous and non-Indigenous children: a population-based cohort study of 100 357 Australian children. *J Epidemiol Community Health* 2021;75(4):371-79.
156. Rao N, Richards B, Sun J, et al. Early childhood education and child development in four countries in East Asia and the Pacific. *EARLY CHILDHOOD RESEARCH QUARTERLY* 2019;47:169-81. doi: doi:10.1016/j.ecresq.2018.08.011
157. Ha S, Yeung E, Bell E, et al. Prenatal and early life exposures to ambient air pollution and development. *Environmental Research* 2019;174:170-75. doi: https://doi.org/10.1016/j.envres.2019.03.064
158. Ahmed SM, Mishra GD, Moss KM, et al. Maternal and Childhood Ambient Air Pollution Exposure and Mental Health Symptoms and Psychomotor Development in Children: An Australian Population-Based Longitudinal Study. *ENVIRONMENT INTERNATIONAL* 2022;158 doi: doi:10.1016/j.envint.2021.107003
159. Shih P, Chiang TL, Wu CD, et al. Air pollution during the perinatal period and neurodevelopment in children: A national population study in Taiwan. *DEVELOPMENTAL MEDICINE AND CHILD NEUROLOGY* 2023;65(6):783-91. doi: doi:10.1111/dmcn.15430
160. Chiu YHM, Hsu HHL, Coull BA, et al. Prenatal particulate air pollution and neurodevelopment in urban children: Examining sensitive windows and sex-specific associations. *ENVIRONMENT INTERNATIONAL* 2016;87:56-65. doi: doi:10.1016/j.envint.2015.11.010
161. Jarvis I, Davis Z, Sbihi H, et al. Assessing the association between lifetime exposure to greenspace and early childhood development and the mediation

effects of air pollution and noise in Canada: a population-based birth cohort study. *LANCET PLANETARY HEALTH* 2021;5(10):E709-E17. doi: doi:
162. Lertxundi A, Baccini M, Lertxundi N, et al. Exposure to fine particle matter, nitrogen dioxide and benzene during pregnancy and cognitive and psychomotor developments in children at 15 months of age. *ENVIRONMENT INTERNATIONAL* 2015;80:33-40. doi: doi:10.1016/j.envint.2015.03.007
163. Odo DB, Yang IA, Dey S, et al. A cross-sectional analysis of long-term exposure to ambient air pollution and cognitive development in children aged 3-4 years living in 12 low- and middle-income countries. *ENVIRONMENTAL POLLUTION* 2023;318 doi: doi:10.1016/j.envpol.2022.120916
164. Porta D, Narduzzi S, Badaloni C, et al. Air pollution and cognitive development at age 7 in a prospective Italian birth cohort. *Epidemiology* 2016;27:228-36. doi: doi:
165. Wang PP, Zhao YY, Li JL, et al. Prenatal exposure to ambient fine particulate matter and early childhood neurodevelopment: A population-based birth cohort study. *SCIENCE OF THE TOTAL ENVIRONMENT* 2021;785 doi: doi:10.1016/j.scitotenv.2021.147334
166. Wang HJ, Zhang HL, Li JX, et al. Prenatal and early postnatal exposure to ambient particulate matter and early childhood neurodevelopment: A birth cohort study. *ENVIRONMENTAL RESEARCH* 2022;210 doi: doi:10.1016/j.envres.2022.112946
167. Kim E, Park H, Hong YC, et al. Prenatal exposure to PM₁₀ and NO₂ and children's neurodevelopment from birth to 24 months of age: Mothers and Children's Environmental Health (MOCEH) study. *SCIENCE OF THE TOTAL ENVIRONMENT* 2014;481:439-45. doi: doi:10.1016/j.scitotenv.2014.01.107
168. Freire C, Ramos R, Puertas R, et al. Association of traffic-related air pollution with cognitive development in children. *JOURNAL OF EPIDEMIOLOGY AND COMMUNITY HEALTH* 2010;64(3):223-28. doi: doi:10.1136/jech.2008.084574
169. Yu T, Zhou LL, Xu J, et al. Effects of prenatal exposures to air sulfur dioxide/nitrogen dioxide on toddler neurodevelopment and effect modification by ambient temperature. *ECOTOXICOLOGY AND ENVIRONMENTAL SAFETY* 2022;230 doi: doi:10.1016/j.ecoenv.2021.113118
170. Bai Y, Shang G, Wang L, et al. The relationship between birth season and early childhood development: Evidence from northwest rural China. *PLoS One* 2018;13(10):e0205281. doi: doi:10.1371/journal.pone.0205281
171. Goto R, Frodl T, Skokauskas N. Armed Conflict and Early Childhood Development in 12 Low- and Middle-Income Countries. *Pediatrics* 2021;148(3) doi: doi:10.1542/peds.2021-050332
172. Bornstein MH, Rothenberg WA, Lansford JE, et al. Child Development in Low- and Middle-Income Countries. *PEDIATRICS* 2021;148(5) doi: doi:10.1542/peds.2021-053180
173. Collier LR, Gregory T, Harman-Smith Y, et al. Inequalities in child development at school entry: A repeated cross-sectional analysis of the Australian Early Development Census 2009–2018. *The Lancet Regional Health–Western Pacific* 2020;4
174. Taylor CL, Christensen D, Venn AJ, et al. Use of administrative record linkage to examine patterns of universal early childhood health and education service use from birth to Kindergarten (age four years) and developmental vulnerability in the Preparatory Year (age five years) in Tasmania, Australia. *International Journal of Population Data Science* 2021;6(3)
175. Chartier MJ, Brownell MD, Isaac MR, et al. Is the Families First Home Visiting Program Effective in Reducing Child Maltreatment and Improving Child Development? *Child Maltreatment* 2017;22(2):121-31. doi: doi:10.1177/1077559517701230
176. Enns JE, Nickel NC, Chartier M, et al. An unconditional prenatal income supplement is associated with improved birth and early childhood outcomes among First Nations children in Manitoba, Canada: a population-based cohort study. *BMC pregnancy and childbirth* 2021;21:1-11.

Table S4. Risk of bias assessment for studies included in the systematic review.

Study ID	First author, Year	Study design	Q1	Q2	Q3	Q4	Q5	Q6	Q7	Q8	Q9	Q10	Overall appraisal
1	Bell, 2018	Cohort	Yes	Yes	Yes	Yes	Yes	Yes	Yes	Yes	Yes	Yes	Low Risk
2	Abubakar, 2010	Cohort	Yes	Yes	Yes	No	No	Yes	Yes	No	Yes	No	High Risk
3	Ahmed, 2022	Cohort	Yes	Yes	Yes	Yes	Yes	Yes	Yes	Yes	Yes	Yes	Low Risk
4	Wang, 2023	Prospective Cohort	Yes	Yes	Yes	No	No	Yes	Yes	Yes	Yes	Yes	Moderate Risk
5	Handal, 2016	Cohort	Yes	Yes	Yes	Yes	Yes	Yes	Yes	Yes	Yes	Yes	Low Risk
6	Allel, 2021	Cross-sectional	Yes	Yes	Yes	Yes	Yes	Yes	Yes	Yes			Low Risk
7	Bai, 2018	Cohort	Yes	Yes	Yes	Yes	Yes	Yes	Yes	Yes	Yes	No	Moderate Risk
8	Ballantyne, 2016	Prospective Cohort	Yes	Yes	Yes	Yes	Yes	Yes	Yes	Yes	Yes	Yes	Low Risk
9	Basnet, 2022	Survey	Yes	Yes	Yes	Yes	Yes	Yes	Yes	Yes			Low Risk
10	Bell, 2016	Cohort	Yes	Yes	Yes	Yes	Yes	Yes	Yes	Yes	Yes	Yes	Low Risk
11	Bell, 2023	Cohort	Yes	Yes	Yes	Yes	Yes	Yes	Yes	Yes	Yes	Yes	Low Risk
12	Berglund, 2017	Cohort	Yes	Yes	Yes	Yes	Yes	Yes	Yes	Yes	Yes	No	Moderate Risk
13	Bornstein, 2021	Cross-sectional	Yes	Yes	Yes	Yes	No	No	Yes	No			High Risk
14	Cao, 2022	Cohort	Yes	Yes	Yes	Yes	Yes	Yes	Yes	Yes	Yes	Yes	Low Risk
15	Chartier,2017	Cohort	Yes	Yes	Yes	No	No	Yes	Yes	Yes	Yes	Yes	Moderate Risk
16	Chaudhury, 2018	Cohort	Yes	Yes	Yes	No	No	Yes	Yes	Yes	Yes	Yes	Low Risk
17	Chen, 2022	Cohort	Yes	Yes	Yes	Yes	Yes	Yes	Yes	Yes	Yes	Yes	Moderate Risk
18	Chen, 2013	Cohort	Yes	Yes	Yes	No	No	No	No	Yes	Yes	Yes	High Risk
19	Cruz-Rodríguez, 2023	Cohort	Yes	Yes	Yes	Yes	Yes	Yes	Yes	Yes	Yes	Yes	Low Risk
20	De Moura, 2010	Cohort	Yes	Yes	Yes	No	No	Yes	Yes	No	Yes	Yes	Moderate Risk
21	Dean,2002	Cohort	Yes	Yes	Yes	No	No	Yes	Yes	Yes	Yes	Yes	Moderate Risk
22	Deave, 2008	Prospective Cohort	Yes	Yes	Yes	Yes	Yes	Yes	Yes	Yes	Yes	Yes	Low Risk
23	Demirci, 2018	Nested case control	Yes	Yes	Yes	Yes	Yes	Yes	Yes	Yes	Yes	Yes	Low Risk
24	Dhamrait, 2021	Cohort	Yes	Yes	Yes	Yes	Yes	Yes	Yes	Yes	Yes	Yes	Low Risk

25	Domingues, 2014	Cohort	Yes	Yes	Yes	Yes	Yes	Yes	Yes	No	Yes	Yes	Moderate Risk
26	Drago, 2020	Cohort	Yes	Yes	Yes	Yes	Yes	Yes	Yes	Yes	Yes	Yes	Low Risk
27	Duko, 2023	Cohort	Yes	Yes	Yes	Yes	Yes	Yes	Yes	Yes	Yes	Yes	Low Risk
28	Duko, 2023	Cohort	Yes	Yes	Yes	Yes	Yes	Yes	Yes	Yes	Yes	Yes	Low Risk
29	Eickmann, 2007	Cohort	Yes	Yes	Yes	Yes	Yes	Yes	Yes	No	Yes	Yes	Low Risk
30	Falster, 2018	Cohort	Yes	Yes	Yes	Yes	Yes	Yes	Yes	Yes	Yes	Yes	Low Risk
31	Fardell, 2023	Cohort	Yes	Yes	Yes	Yes	Yes	Yes	Yes	Yes	Yes	Yes	Low Risk
32	Flick, 2011	Cohort	Yes	Yes	Yes	No	No	Yes	Yes	Yes	Yes	Yes	Moderate Risk
33	Freire, 2010	Cohort	Yes	Yes	Yes	Yes	Yes	Yes	Yes	Yes	Yes	Yes	Low Risk
34	Gao, 2021	Cross-sectional	Yes	Yes	Yes	Yes	Yes	Yes	Yes	No			
35	Ghassabian, 2016	Cohort	Yes	Yes	Yes	Yes	Yes	Yes	Yes	Yes	Yes	Yes	Low Risk
36	Gleason, 2021	Cohort	Yes	Yes	Yes	Yes	Yes	Yes	Yes	Yes	Yes	Yes	Low Risk
37	Gonzalez, 2017	Prospective Cohort	Yes	Yes	Yes	Yes	Yes	Yes	Yes	Yes	Yes	Yes	Low Risk
38	Goto, 2021	Cross-sectional	Yes	Yes	Yes	Yes	Yes	Yes	Yes	Yes			Low Risk
39	Graham, 2016	Cohort	Yes	Yes	Yes	No	No	Yes	Yes	Yes	Yes	Yes	Moderate Risk
40	Green, 2018	Cohort	Yes	Yes	Yes	Yes	Yes	Yes	Yes	Yes	Yes	Yes	Low Risk
41	Grippo, 2023	Cohort	Yes	Yes	Yes	Yes	Yes	Yes	Yes	Yes	Yes	Yes	Low Risk
42	Gül, 2020	Cohort	Yes	Yes	Yes	No	No	Yes	Yes	NO	Yes	Yes	High Risk
43	Guo, 2019	Cohort	Yes	Yes	Yes	Yes	Yes	Yes	Yes	Yes	Yes	Yes	Low Risk
44	Halpern, 2009	Cohort	Yes	Yes	Yes	No	No	Yes	Yes	Yes	Yes	Yes	Moderate Risk
45	Hanly, 2018	Cohort	Yes	Yes	Yes	Yes	Yes	Yes	Yes	Yes	Yes	Yes	Low Risk
46	Hao, 2022	Cohort	Yes	Yes	Yes	Yes	Yes	Yes	Yes	Yes	Yes	Yes	Low Risk
47	Hasan, 2022	Survey	Yes	Yes	Yes	Yes	Yes	Yes	Yes	Yes			Low Risk
48	He, 2018	Cross-sectional	Yes	Yes	Yes	Yes	Yes	Yes	Yes	Yes			Low Risk
49	Hinkle, 2012	Cohort	Yes	Yes	Yes	Yes	Yes	Yes	Yes	No	Yes	Yes	Moderate Risk
50	HOLST, 2015	Cohort	Yes	Yes	Yes	Yes	Yes	Yes	Yes	Yes	Yes	Yes	Low Risk
51	Horton, 2011	Cohort	Yes	Yes	Yes	Yes	Yes	Yes	Yes	Yes	Yes	Yes	Low Risk
52	Hua, 2022	Cohort	Yes	Yes	Yes	Yes	Yes	Yes	Yes	Yes	Yes	Yes	Low Risk
53	Huang, 2014	Cohort	Yes	Yes	Yes	Yes	Yes	Yes	Yes	Yes	Yes	Yes	Low Risk
54	Ibanez, 2015	Cohort	Yes	Yes	Yes	Yes	Yes	Yes	Yes	Yes	Yes	Yes	Low Risk

55	Islam, 2023	Survey	Yes	Yes	Yes	Yes	Yes	Yes	Yes	Yes			Low Risk
56	Jang, 2021	Cohort	Yes	Yes	Yes	Yes	Yes	Yes	Yes	No	Yes	Yes	Moderate Risk
57	Jarvis, 2021	Cohort	Yes	Yes	Yes	Yes	Yes	Yes	Yes	Yes	Yes	Yes	Low Risk
58	Jedrychowski, 2012	Cohort	Yes	Yes	Yes	Yes	Yes	Yes	Yes	Yes	Yes	Yes	Low Risk
59	Jeong, 2020	Survey	Yes	Yes	Yes	Yes	Yes	Yes	Yes	Yes			Low Risk
60	Julvez, 2007	Cohort	Yes	Yes	Yes	Yes	Yes	Yes	Yes	Yes	Yes	Yes	Low Risk
61	Junge, 2017	Cohort	Yes	Yes	Yes	Yes	Yes	Yes	Yes	No	Yes	Yes	Low Risk
62	Kariuki, 2016	Cohort	Yes	Yes	Yes	Yes	Yes	Yes	Yes	No	Yes	Yes	Low Risk
63	Keim, 2011	Cohort	Yes	Yes	Yes	Yes	Yes	Yes	Yes	Yes	Yes	Yes	Low Risk
64	Kennedy, 2023	Cohort	Yes	Yes	Yes	Yes	Yes	Yes	Yes	Yes	Yes	Yes	Low Risk
65	Kerstjens, 2011	Prospective Cohort	Yes	Yes	Yes	Yes	Yes	Yes	Yes	Yes	Yes	Yes	Low Risk
66	Kim, 2014	Prospective Cohort	Yes	Yes	Yes	Yes	Yes	Yes	Yes	No	Yes	Yes	Moderate Risk
67	Kim, 2023	Cohort	Yes	Yes	Yes	Yes	Yes	Yes	Yes	Yes	Yes	Yes	Low Risk
68	Le˜ao, 2023	Cohort	Yes	Yes	Yes	Yes	Yes	Yes	Yes	No	Yes	Yes	Moderate Risk
69	Leonard, 2020	Cohort	Yes	Yes	Yes	Yes	Yes	Yes	Yes	Yes	Yes	Yes	Low Risk
70	Lertxundi, 2019	Cohort	Yes	Yes	Yes	Yes	Yes	Yes	Yes	Yes	Yes	Yes	Low Risk
71	Lertxundi, 2015	Cohort	Yes	Yes	Yes	Yes	Yes	Yes	Yes	Yes	Yes	Yes	Low Risk
72	Liu, 2015	Cohort	Yes	Yes	Yes	No	No	Yes	Yes	Yes	Yes	Yes	Moderate Risk
73	Luo, 2015	Cohort	Yes	Yes	Yes	No	No	Yes	Yes	Yes	Yes	Yes	Moderate Risk
74	Ma, 2021	Cohort	Yes	Yes	Yes	Yes	Yes	Yes	Yes	Yes	Yes	Yes	Low Risk
75	Ma, 2022	Cohort	Yes	Yes	Yes	No	No	Yes	Yes	Yes	Yes	Yes	Moderate Risk
76	Burger, 2022	Cohort	Yes	Yes	Yes	Yes	Yes	Yes	Yes	Yes	Yes	Yes	Low Risk
77	McCormack, 2018	Cohort	Yes	Yes	Yes	Yes	Yes	Yes	Yes	Yes	Yes	Yes	Low Risk
78	Mensah, 2010	Cohort	Yes	Yes	Yes	Yes	Yes	Yes	Yes	Yes	Yes	No	Moderate Risk
79	Mora, 2018	Cohort	Yes	Yes	Yes	Yes	Yes	Yes	Yes	Yes	Yes	Yes	Low Risk
80	Moreno-Giménez, 2022	Cohort	Yes	Yes	Yes	No	No	Yes	Yes	Yes	Yes	Yes	Moderate Risk
81	MORROW, 2003	Cohort	Yes	Yes	Yes	Yes	No	No	Yes	Yes	Yes	Yes	Moderate Risk
82	Neamah, 2018	Survey	Yes	Yes	Yes	Yes	Yes	Yes	Yes	Yes			Low Risk
83	Motoki, 2022	Cohort	Yes	Yes	Yes	Yes	Yes	Yes	Yes	Yes	Yes	Yes	Low Risk

84	NELSON, 2004	Cohort	Yes	Yes	Yes	Yes	Yes	Yes	Yes	Yes	Yes	No	Moderate Risk
85	O’Leary, 2019	Cohort	Yes	Yes	Yes	No	No	Yes	Yes	Yes	Yes	Yes	Moderate Risk
86	Odo, 2023	Survey	Yes	Yes	Yes	Yes	No	Yes	Yes	Yes			
87	O’Neill, 2017	Cohort	Yes	Yes	Yes	No	No	Yes	Yes	Yes	Yes	Yes	Moderate Risk
88	Ozkan, 2012	Cohort	Yes	Yes	Yes	Yes	Yes	Yes	Yes	No	Yes	Yes	Low Risk
89	Perera, 2006	Cohort	Yes	Yes	Yes	Yes	Yes	Yes	Yes	No	Yes	Yes	Moderate Risk
90	Pokharel, 2023	Cohort	Yes	Yes	Yes	No	No	Yes	Yes	Yes	Yes	No	Moderate Risk
91	Polanska, 2017	Cohort	Yes	Yes	Yes	Yes	Yes	Yes	Yes	Yes	Yes	No	Moderate Risk
92	Polte, 2019	Cohort	Yes	Yes	Yes	Yes	Yes	Yes	Yes	Yes	Yes	Yes	Low Risk
93	Porta, 2016	Cohort	Yes	Yes	Yes	Yes	Yes	Yes	Yes	Yes	Yes	Yes	Low Risk
94	Quevedo, 2012	Cohort	Yes	Yes	Yes	Yes	Yes	Yes	Yes	Yes	Yes	Yes	Low Risk
95	Ramchandani, 2005	Cohort	Yes	Yes	Yes	Yes	Yes	Yes	Yes	Yes	Yes	Yes	Low Risk
96	Rana, 2022	Survey	Yes	Yes	Yes	Yes	Yes	Yes	Yes	Yes			Low Risk
97	Rao, 2019	Survey	Yes	Yes	Yes	Yes	Yes	No	Yes	Yes			
98	Reardon, 2023	Cohort	Yes	Yes	Yes	No	No	Yes	Yes	Yes	Yes	Yes	Moderate Risk
99	Richards, 2016	Cohort	Yes	Yes	Yes	No	No	Yes	Yes	Yes	Yes	Yes	Moderate Risk
100	Rithipukdee, 2022	Cross-sectional	Yes	Yes	Yes	Yes	Yes	Yes	Yes	Yes			Low Risk
101	Rivero, 2023	Cross-sectional	Yes	Yes	Yes	Yes	Yes	Yes	Yes	No			
102	Rocha, 2022	Cross-sectional	Yes	Yes	Yes	Yes	Yes	Yes	Yes	Yes			Low Risk
103	Rocha, 2022	Cross-sectional	Yes	Yes	Yes	Yes	Yes	Yes	Yes	Yes			Low Risk
104	Rocha, 2022	Cross-sectional	Yes	Yes	Yes	Yes	Yes	Yes	Yes	Yes			Low Risk
105	Rogers, 2023	Cohort	Yes	Yes	Yes	Yes	Yes	Yes	Yes	Yes	Yes	Yes	Low Risk
106	Rohde, 2023	Cohort	Yes	Yes	Yes	Yes	Yes	Yes	Yes	Yes	Yes	No	Moderate Risk
107	ROUX, 2018	Cohort	Yes	Yes	Yes	Yes	Yes	Yes	Yes	Yes	Yes	Yes	Low Risk
108	Roy, 2022	Cohort	Yes	Yes	Yes	No	No	Yes	Yes	No	Yes	Yes	Moderate Risk
109	Russell, 2022	Cross-sectional study	Yes	Yes	Yes	Yes	Yes	Yes	Yes	Yes			Low Risk
110	Saha, 2010	Cohort	Yes	Yes	Yes	Yes	Yes	Yes	Yes	Yes	Yes	Yes	Low Risk
111	Saleem, 2014	Cohort	Yes	Yes	Yes	Yes	Yes	Yes	Yes	Yes	Yes	Yes	Low Risk
112	Sansavini, 2011	Cohort	Yes	Yes	Yes	Yes	Yes	Yes	Yes	Yes	Yes	Yes	Low Risk

113	Shih, 2023	Cohort	Yes	Yes	Yes	No	No	Yes	Yes	Yes	Yes	Yes	Moderate Risk
114	Shrestha, 2022	Cross-sectional study	Yes	Yes	Yes	Yes	Yes	Yes	Yes	Yes			Low Risk
115	Shuffrey, 2022	Cohort	Yes	Yes	Yes	Yes	Yes	Yes	Yes	Yes	Yes	Yes	Low Risk
116	Slykerman, 2007	Cross-sectional study	Yes	Yes	Yes	Yes	Yes	Yes	Yes	Yes			Low Risk
117	Smith, 2023	Cohort	Yes	Yes	Yes	No	No	Yes	Yes	Yes	Yes	Yes	Moderate Risk
118	Smith-Nielsen, 2019	Cohort	Yes	Yes	Yes	No	No	Yes	Yes	No	Yes	Yes	High Risk
119	Smithers, 2015	Cohort	Yes	Yes	Yes	Yes	Yes	Yes	Yes	No	Yes	Yes	Moderate Risk
120	Strobel, 2020	Cohort	Yes	Yes	Yes	Yes	Yes	Yes	Yes	Yes	Yes	Yes	Low Risk
121	Gupta, 2021	Cross-sectional study	Yes	Yes	Yes	Yes	Yes	Yes	Yes	Yes			Low Risk
122	Syrengelas, 2014	Cross-sectional study	Yes	Yes	Yes	Yes	Yes	Yes	Yes	Yes			Low Risk
123	Tao, 2022	Cohort	Yes	Yes	Yes	Yes	Yes	Yes	Yes	Yes	Yes	Yes	Low Risk
124	Tran, 2013	Cohort	Yes	Yes	Yes	Yes	Yes	Yes	Yes	Yes	Yes	Yes	Low Risk
125	Tofail, 2009	Cohort	Yes	Yes	Yes	No	No	Yes	Yes	Yes	Yes	No	High Risk
126	Trumpff, 2015	Cohort	Yes	Yes	Yes	No	No	Yes	Yes	Yes	Yes	No	High Risk
127	Turunç, 2022	Survey	Yes	Yes	Yes	Yes	Yes	No	Yes	No			
128	Urizar, 2022	Cohort	Yes	Yes	Yes	No	Yes	Yes	Yes	Yes	Yes	Yes	Moderate Risk
129	Utami, 2023	Cohort	Yes	Yes	Yes	No	Yes	Yes	Yes	Yes	Yes	Yes	Moderate Risk
130	Van der Veere,- 2020	Cohort	Yes	Yes	Yes	No	No	Yes	Yes	Yes	Yes	Yes	Moderate Risk
131	Varadarajan, 2021	Cross-sectional study	Yes	Yes	Yes	Yes	No	Yes	Yes	Yes			
132	Vrijheid, 2012	Cohort	Yes	Yes	Yes	Yes	Yes	Yes	Yes	Yes	Yes	Yes	Low Risk
133	Wall-Wieler, 2020	Cohort	Yes	Yes	Yes	No	No	Yes	Yes	Yes	Yes	Yes	Moderate Risk
134	Wallenborn, 2021	Cohort	Yes	Yes	Yes	Yes	Yes	Yes	Yes	Yes	Yes	Yes	Low Risk
135	Wang, 2022	Cohort	Yes	Yes	Yes	Yes	Yes	Yes	Yes	Yes	Yes	Yes	Low Risk
136	Wang, 2019	Cross-sectional study	Yes	Yes	Yes	Yes	Yes	No	Yes	Yes			
137	Wang, 2021	Cohort	Yes	Yes	Yes	Yes	Yes	Yes	Yes	Yes	Yes	Yes	Low Risk
138	Warshafsky, 2016	Prospective Cohort	Yes	Yes	Yes	Yes	Yes	Yes	Yes	Yes	Yes	Yes	Low Risk
139	Webb, 2020	Cohort	Yes	Yes	Yes	Yes	Yes	Yes	Yes	Yes	Yes	Yes	Low Risk
140	Wehby, 2011	Cohort	Yes	Yes	Yes	No	No	Yes	Yes	Yes	Yes	Yes	Moderate Risk

141	Wei, 2021	Cohort	Yes	Yes	Yes	Yes	Yes	Yes	Yes	Yes	Yes	Yes	Low Risk
142	Widen, 2019	Cohort	Yes	Yes	Yes	Yes	Yes	Yes	Yes	Yes	Yes	Yes	Low Risk
143	Wu, 2018	Cohort	Yes	Yes	Yes	No	No	Yes	Yes	Yes	Yes	Yes	Moderate Risk
144	Yu, 2022	Cohort	Yes	Yes	Yes	Yes	Yes	Yes	Yes	No	Yes	Yes	Low Risk
145	Zheng, 2021	Cohort	Yes	Yes	Yes	No	No	Yes	Yes	Yes	Yes	Yes	Moderate Risk
146	Zhong, 2021	Cohort	Yes	Yes	Yes	Yes	Yes	Yes	Yes	Yes	Yes	Yes	Low Risk
147	Bin, 2017	Cohort	Yes	Yes	Yes	Yes	Yes	Yes	Yes	Yes	Yes	Yes	Low Risk
148	Brinkman, 2012	Census	Yes	Yes	Yes	Yes	Yes	Yes	Yes	Yes			Low Risk
149	Christian, 2017	Census	Yes	Yes	Yes	Yes	Yes	Yes	Yes	Yes			Low Risk
150	Collier, 2020	Census	Yes	Yes	Yes	Yes	Yes	Yes	Yes	Yes			Low Risk
151	Crockett, 2022	Cohort	Yes	Yes	Yes	Yes	Yes	Yes	Yes	Yes	Yes	Yes	Low Risk
152	Curtin, 2013	Cross-sectional study	Yes	Yes	Yes	Yes	Yes	Yes	Yes	Yes			Low Risk
153	Dhamrait, 2022	Cohort	Yes	Yes	Yes	Yes	Yes	Yes	Yes	Yes	Yes	Yes	Low Risk
154	Dhamrait, 2021	Cohort	Yes	Yes	Yes	Yes	Yes	Yes	Yes	Yes	Yes	Yes	Low Risk
155	Enns, 2021	Cohort	Yes	Yes	Yes	Yes	Yes	Yes	Yes	Yes	Yes	Yes	Low Risk
156	Falster, 2020	Cohort	Yes	Yes	Yes	Yes	Yes	Yes	Yes	Yes	Yes	Yes	Low Risk
157	Ha, 2019	Cohort	Yes	Yes	Yes	Yes	Yes	Yes	Yes	Yes	Yes	Yes	Low Risk
158	Janus, 2019	Cohort	Yes	Yes	Yes	Yes	Yes	Yes	Yes	Yes	Yes	Yes	Low Risk
159	Lindquist, 2022	Cohort	Yes	Yes	Yes	Yes	Yes	Yes	Yes	Yes	Yes	Yes	Low Risk
160	Morris, 2021	Cohort	Yes	Yes	Yes	Yes	Yes	Yes	Yes	Yes	Yes	Yes	Low Risk
161	O'Leary, 2016	Cohort	Yes	Yes	Yes	Yes	Yes	Yes	Yes	Yes	Yes	Yes	Low Risk
162	Pearce, 2016	Cohort	Yes	Yes	Yes	Yes	Yes	Yes	Yes	Yes	Yes	Yes	Low Risk
163	Razaz, 2016	Cohort	Yes	Yes	Yes	Yes	Yes	Yes	Yes	Yes	Yes	Yes	Low Risk
164	Razaz, 2019	Cohort	Yes	Yes	Yes	Yes	Yes	Yes	Yes	Yes	Yes	Yes	Low Risk
165	Razaz, 2016	Cohort	Yes	Yes	Yes	Yes	Yes	Yes	Yes	Yes	Yes	Yes	Low Risk
166	Simpson, 2020	Cohort	Yes	Yes	Yes	Yes	Yes	Yes	Yes	Yes	Yes	Yes	Low Risk
167	Singal, 2020	Cohort	Yes	Yes	Yes	Yes	Yes	Yes	Yes	Yes	Yes	Yes	Low Risk
168	Taylor, 2020	Cohort	Yes	Yes	Yes	Yes	Yes	Yes	Yes	Yes	Yes	Yes	Low Risk
169	Taylor, 2021	Cohort	Yes	Yes	Yes	Yes	Yes	Yes	Yes	Yes	Yes	Yes	Low Risk
170	Whitten, 2022	Cohort	Yes	Yes	Yes	Yes	Yes	Yes	Yes	Yes	Yes	Yes	Low Risk

171	Williamson, 2019	Cohort	Yes	Yes	Yes	Yes	Yes	Yes	Yes	Yes	Yes	Yes	Low Risk
172	Duko, 2024	Cohort	Yes	Yes	Yes	Yes	Yes	Yes	Yes	Yes	Yes	Yes	Low Risk
173	Kerai, 2022	Cohort	Yes	Yes	Yes	Yes	Yes	Yes	Yes	Yes	Yes	Yes	Low Risk
174	Peterson, 2019	Cohort	Yes	Yes	Yes	Yes	Yes	Yes	Yes	Yes	Yes	Yes	Low Risk
175	Smithers, 2015	Cohort	Yes	Yes	Yes	Yes	Yes	Yes	Yes	Yes	Yes	Yes	Low Risk
